# Epiblast-like stem cells established by Wnt/β-catenin signaling manifest distinct features of formative pluripotency and germline competence

**DOI:** 10.1016/j.celrep.2023.112021

**Published:** 2023-01-23

**Authors:** Qing Luo, Han-pin Pui, Jiayu Chen, Leqian Yu, Paulo R. Jannig, Yu Pei, Linxuan Zhao, Xingqi Chen, Sophie Petropoulos, Jorge L. Ruas, Jun Wu, Qiaolin Deng

**Affiliations:** 1 Department of Physiology and Pharmacology, Karolinska Institutet, 171 77 Stockholm, Sweden; 2 Center for Molecular Medicine, Karolinska University Hospital, 171 77 Stockholm, Sweden; 3 Division of Obstetrics and Gynecology, Department of Clinical Science, Intervention and Technology, Karolinska Institutet and Karolinska University Hospital, 141 52 Huddinge, Sweden; 4 Clinical and Translation Research Center of Shanghai First Maternity & Infant Hospital, Shanghai Key Laboratory of Signaling and Disease Research, School of Life Sciences and Technology, Tongji University, Shanghai 20092, China; 5 Frontier Science Center for Stem Cell Research, Tongji University, Shanghai 20092, China; 6 Department of Molecular Biology, University of Texas Southwestern Medical Center, Dallas, TX 75390, USA; 7 Hamon Center for Regenerative Science and Medicine, University of Texas Southwestern Medical Center, Dallas, TX 75390, USA; 8 Cecil H. and Ida Green Center for Reproductive Biology Sciences, University of Texas Southwestern Medical Center, Dallas, TX 75390, USA; 9 Department of Medicine, Centre de recherche du CHUM, University of Montreal, Montreal, QC H2X 0A9, Canada; 10 Department of Immunology, Genetics and Pathology, Science for Life Laboratory, Uppsala University, 751 85 Uppsala, Sweden; 11 These authors contributed equally; 12 Lead contact

## Abstract

Different formative pluripotent stem cells harboring similar functional properties have been recently established to be lineage neutral and germline competent yet have distinct molecular identities. Here, we show that WNT/β-catenin signaling activation sustains transient mouse epiblast-like cells as epiblast-like stem cells (EpiLSCs). EpiLSCs display metastable formative pluripotency with bivalent cellular energy metabolism and unique transcriptomic features and chromatin accessibility. We develop single-cell stage label transfer (scSTALT) to study the formative pluripotency continuum and reveal that EpiLSCs recapitulate a unique developmental period *in vivo*, filling the gap of the formative pluripotency continuum between other published formative stem cells. WNT/β-catenin signaling activation counteracts differentiation effects of activinA and bFGF by preventing complete dissolution of naive pluripotency regulatory network. Moreover, EpiLSCs have direct competence toward germline specification, which is further matured by an FGF receptor inhibitor. Our EpiLSCs can serve as an *in vitro* model for mimicking and studying early post-implantation development and pluripotency transition.

## In brief

Luo et al. report mouse epiblast-like stem cells (EpiLSCs) displaying a metastable formative pluripotency and recapitulating a particular developmental period. EpiLSCs serve as an *in vitro* model for post-implantation development and pluripotency transition.

## Graphical abstract

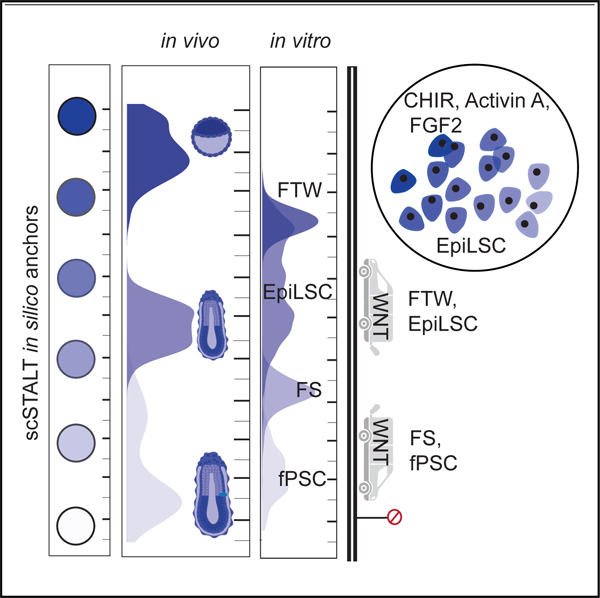

## INTRODUCTION

Pluripotency continuum is restricted to a brief window in early embryonic development during which epiblast cells maintain the plasticity to adopt multiple cell fates. Since the first transient developmental entity was suspended *in vitro*,^1,2^ naive and primed pluripotency from model organisms and humans have been captured with distinct transcriptional,^3–6^ epigenetic (i.e., chromatin and DNA modifications),^7–10^ and metabolic^11–13^ profiles. Above all, chimera contribution and germline competence represent the key functional distinction between them. Notably, pluripotency transition *in vivo* is continuous, therefore the capture of two pluripotent states *in vitro* does not recapitulate the full spectrum of developmental progression, and additional intermediate states were hypothesized to reconcile the direct competence for germline induction and to remain lineage neutral.^14–17^ Epiblast-like cells (EpiLCs), a transient culture with bFGF and activin A render mouse embryonic stem cells (ESCs) some characteristic features of formative pluripotency.^14,16^ Recently, FTW-ESCs,^18^ formative stem (FS) cells,^19^ and fPSCs^20^ are derived from mouse embryos^18,19^ and/or mouse naive ESCs,^20^ exhibiting entire functional properties of formative pluripotency yet differing in WNT/β-catenin signaling modulation either by activation or inhibition.

Our previous study revealed elevated WNT/β-catenin signaling activities at the posterior epiblast, which potentiated these cells to primordial germ cell (PGC) specification.^21^ To further understand the effects of WNT/β-catenin signaling on the pluripotency continuum and characterize the molecular properties toward PGC-like cell (PGCLC) generation, we cultured the germline reporter BVSC (Prdm1-mVenus::Dppa3-ECFP) mouse ESC^22,23^ in a feeder-free condition supplemented with bFGF, activin A, and CHIR99021 similar as previously used for chimeras potentiation.^17^ Interestingly, this culture condition sustained EpiLCs, so they were named epiblast-like stem cells (EpiLSCs). We performed assay for transposase-accessible chromatin with sequencing (ATAC-seq) and single-cell Smart-seq3 on EpiLSCs and compared them with recently published mouse formative pluripotent stem cells (PSCs). Our analyses revealed a distinct chromatin accessibility landscape of EpiLSCs for pluripotency genes and super-bivalent genes. WNT/β-catenin signaling sustains three dynamic cell states in EpiLSCs and maintains the formative pluripotency bridging between FTW and FS cells revealed by our developed pseudotime integration algorithm, single-cell stage label transfer (scSTALT). As a result, EpiLSCs uniquely recapitulate the pluripotent transition of mouse embryonic day (E) 5–6. Different from FTW cells grown on the feeder with WNT/β-catenin signaling, EpiLSCs demonstrate metastable formative pluripotency with some cells toward spontaneous differentiation. However, in contrast to FS cells that primarily utilize glycolysis as energy supply and have lost the potential to form naive pluripotency, EpiLSCs favor bivalent energy metabolism and can adapt to 2i+LIF naive pluripotency culture like FTW cells. We also reconciled the role of WNT/β-catenin signaling in a context-dependent manner to regulate formative pluripotency. Moreover, we identified that the FGF receptor inhibitor PD173074 (PD) not only enhances the PGCLC differentiation efficiency but also contributes to their maturation by upregulation of meiotic cell-cycle genes. Together, our findings provide insights into the formative pluripotency continuum defined by distinct molecular profiles and WNT/β-catenin signaling activity and serve as a unique *in vitro* model for mimicking and studying early post-implantation development and pluripotency transition.

## RESULTS

### Activation of WNT/β-catenin signaling by CHIR sustains EpiLCs with distinct metabolic property

**Figure 1.**
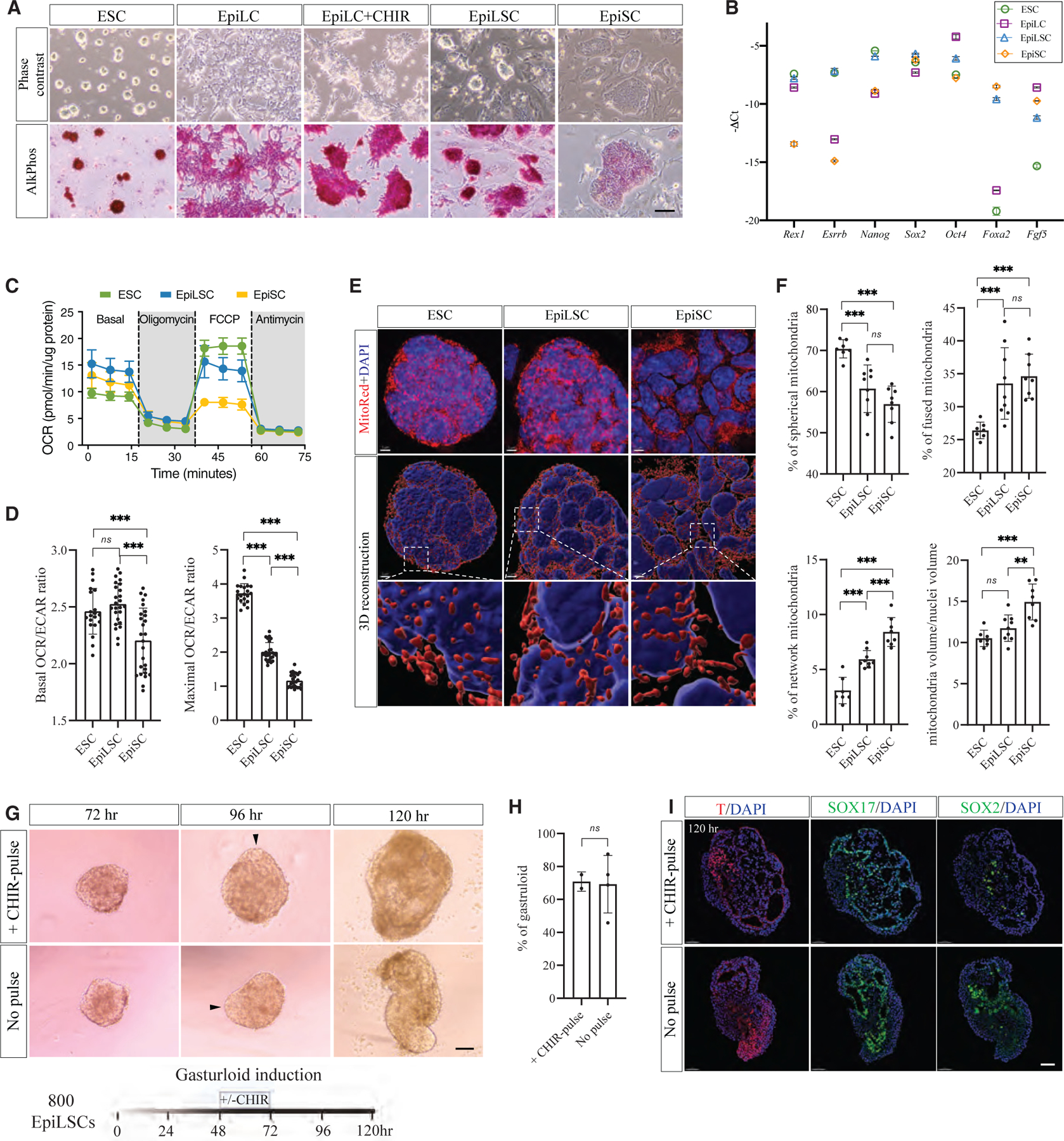
Activation of WNT signaling by CHIR propagates and sustains EpiLCs as EpiLSCs with distinguished metabolism and differentiation potential (A) Morphology of ESCs, day 2 EpiLCs, EpiLCs+CHIR (day 2), EpiLSCs, and EpiSCs shown by phase contrast and AP staining images. Scale bar, 100 μm. (B) qRT-PCR in ESCs, day 2 EpiLCs, EpiLSCs, and EpiSCs. (C and D) Extracellular flux analysis (Seahorse) of cellular respiration and OCR/ECAR ratio under basal and maximal conditions (D) in ESCs, EpiLSCs, and EpiSCs and (D) OCR/ECAR ratio under basal and maximal conditions. Error bars represent SD, ***p < 0.001; two-tailed Student’s t test. (E) Immunofluorescent staining (top) and Imaris 3D renderings (bottom) of mitochondria in ESCs, EpiLSCs, and EpiSCs with MitoTracker Red. Nuclei were counter-stained with DAPI. Scale bar, 5 μm. (F) Quantitative analyses of mitochondrial morphology and relative mitochondria volume. Error bars represent SD; ***p < 0.001, **p < 0.01; two-tailed Student’s t test. (G) Bright-field images of EpiLSCs undergoing gastruloid assay using 800 cells with/without CHIR pulse. Arrowheads indicate protrusion zone. Scale bar, 100 μm. (H) The percentage of EpiLSC embryoid bodies with symmetry breaking with/without CHIR pulse at 120 h. Error bars represent SD; ns: non-significance by two-tailed Student’s t test. (I) Immunostaining of 120 h gastruloid derived from EpiLSCs using 800 cells with/without CHI -pulse at 48 h. Mesodermal, endodermal, and ectodermal cells are distinguished by T, SOX17, and SOX2 expression, respectively. Nuclei were counterstained with DAPI. Scale bar, 50 μm. See also Figure S1.

Previously, we showed that the epiblast regionalization is accompanied by pluripotency transition,^21^ and the posterior epiblast competent for PGC specification expresses high levels of *Wnt3* and its downstream targets *Lef1*, *Tcf7l1*, and *Tcf7l2* (Figure S1A). Meanwhile, compared with naive ESCs, the expression of *Wnt3a* together with its downstream targets was initiated in mouse day 2 EpiLCs (Figure S1B). Given that the role of WNT/β-catenin signaling in regulating different pluripotency states is context dependent, we decided to supplement established EpiLC culture with 3 mM CHIR99021, followed by more than 10 passages (Figure S1C) to investigate whether activating WNT/β-catenin signaling together with priming growth factors would sustain the transient property of EpiLC as EpiLSCs (Figure S1C). We observed that when treated with CHIR99021 for 2 days, EpiLCs+CHIR cells started to aggregate and became more apparent after 10 passages with the formation of irregular dome-shaped colonies and higher alkaline phosphatase (AP) staining in contrast to the flat EpiLCs and EpiSCs (Figure 1A). We next examined the expression of naive (*Rex1* and *Esrrb*), core (*Nanog*, *Sox2*, and *Oct4*), and primed (*Foxa2* and *Fgf5*) pluripotency genes using qPCR. All cells express comparable levels of core pluripotency genes, but EpiLSCs express high levels of both naive and primed pluripotency genes (Figure 1B). In addition, EpiLSCs still maintained normal karyotype after 20 passages (Figure S1D).

A switch from bivalent metabolism in naive state to predominantly glycolytic metabolism in primed state was reported.^12,13,24^ Therefore, we performed the Seahorse metabolic flux assay on ESCs, EpiLSCs, and EpiSCs. Oxygen consumption rate showed that EpiLSCs had an intermediate level of maximal mitochondrial activity between ESCs and EpiSCs (Figure 1C). Analysis of the oxygen consumption rate (OCR)/extracellular acidification rate (ECAR) ratio showed that EpiLSCs utilized oxidative phosphorylation during basal respiration but switched to glycolysis during maximal respiration (Figure 1D). In response to metabolic changes, mitochondria undergo fusion and fission dynamics. We therefore examined mitochondrial morphology by 3D reconstruction and classified three categories based on the sphericity index (Figures 1E and S1E). Notably, EpiLSCs had intermediate spherical (60.7% ± 5.5%) and fused (33.5% ± 5.1%) mitochondrial morphology, whereas ESCs had predominantly spherical mitochondria (70.4% ± 2.1%) and EpiSCs showed the least spherical mitochondria (56.9% ± 4.1%) (Figures 1E and 1F). Furthermore, the mitochondrial volume of EpiLSCs was found comparable to ESCs but less than EpiSCs (Figure 1F).

Gastruloid formation efficiency is greatly reduced from naive to primed PSCs.^25^ Hence, we used gastruloid formation to evaluate pluripotency status of EpiLSCs.^26,27^ We observed that when starting with 300 cells, EpiLSCs failed to form gastruloid (Figure S1F). When starting with 800 cells, we observed the formation of elongated structure from aggregates at 96 h with or without a CHIR pulse, which was more obvious at 120 h (Figures 1G and 1H). To confirm the specification of three germ layers in gastruloids, we examined expression of T (a mesoderm marker) and SOX17 (an endoderm marker) as well as SOX2 (an ectoderm marker) and detected a characteristic polarized T expression in the protrusion (Figure 1I). We further examined the differentiation efficiency of EpiLSCs into somatic cell fate. In response to mesoderm induction, we observed upregulation of mesodermal marker *T* and *Eomes* expression, which was confirmed by T^+^ cells at day 3 (Figure S1G). Similarly, endodermal marker gene expression of *Sox17* and *Foxa2* as well as SOX17^+^ cells at day 3 were detected when EpiLSCs were exposed to endodermal differentiation condition (Figure S1H). Lastly, in response to neural lineage induction, we also observed upregulation of neural marker genes *Sox1* and *Tubb3* together with TUBB3^+^ cells at day 3 (Figure S1I). Taken together, these results showed that EpiLSCs derived by the propagation of EpiLC with CHIR supplementation are distinct from ESCs and EpiSCs. EpiLSCs are pluripotent, utilize bivalent respiration, and retain the lineage-neutral capacity.

### EpiLSCs show distinct chromatin accessibility associated with formative pluripotency and germline potency

**Figure 2.**
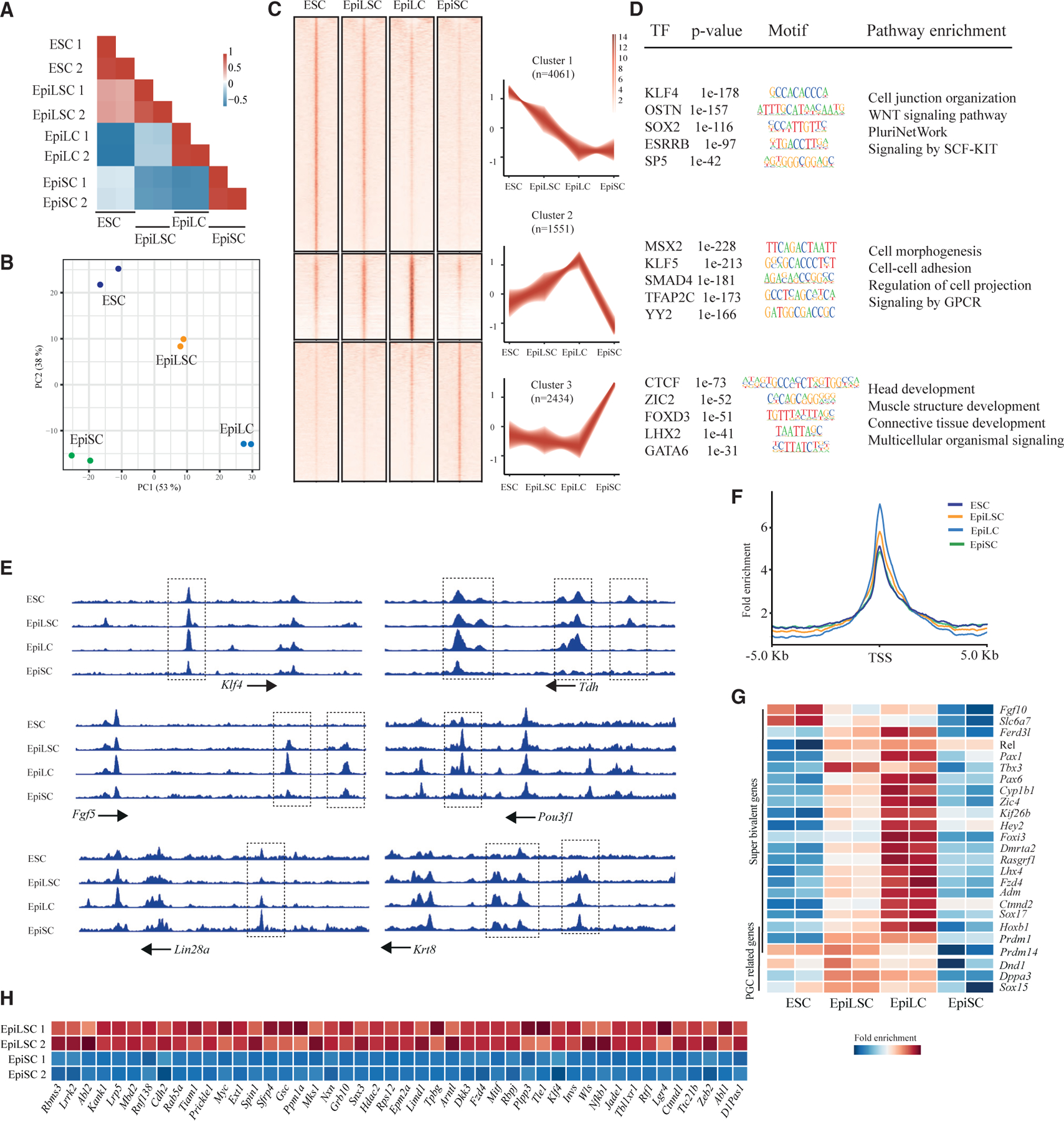
EpiLSCs show distinct chromatin accessibility associated with formative pluripotency and germline potency (A) Correlation heatmap of ESCs, EpiLSCs, EpiLCs, and EpiSCs. Label 1 and 2 means two replicates. (B) PCA plot of ESCs, EpiLSCs, EpiLCs, and EpiSCs. Each cell line has two replicates as shown with the two dots. (C) Left: heatmap of the three clusters of differential peaks in ESCs, EpiLSCs, EpiLCs, and EpiSCs. 2 kb flanking regions were included. Right: scaled value of the peaks showing different trend in the three clusters; n stands for peak number. (D) Enriched motif and pathway of the three clusters of differential peaks. (E) Track plot of ATAC-seq openness at the selected loci. (F) Average openness of the TSS regions of super-bivalent genes. Flanking regions of 10 kb were included. (G) Heatmap of scaled ATAC-seq normalized counts in the TSS regions of the selected genes. The genes were annotated as super-bivalent and PGC-related genes. (H) Heatmap of scaled ATAC-seq normalized counts of EpiLSCs and EpiSCs in TSS regions of Wnt pathway-related genes. See also Figure S2 and Table S1.

To reveal the chromatin accessibility of EpiLSCs, we performed the ATAC-seq^28^ of EpiLSCs compared with ESCs, EpiLCs, and EpiSCs^29^ (Figure S2A). Pearson correlation analysis showed that EpiLSCs are most similar to ESCs, followed by EpiLCs (Figure 2A). Similarly, principal-component analysis (PCA) suggested the transition along the naive-to-primed pluripotent continuum with EpiLSCs positioned between ESCs and EpiLCs (Figure 2B). To further elucidate the chromatin global changes, we identified three clusters from differentially accessible peaks (Figure 2C). Transcription factor (TF) binding motif enrichment revealed that cluster 1, 2, and 3 signatured naive (e.g., KLF4, OSTN, and ESRRB), formative (e.g., MSX2, KLF5, and SMAD4), and primed (e.g., germline fate inhibitor FOXD3,^30^ ZIC2, and GATA6) pluripotency related TFs, respectively (Figure 2D; Table S1). Accordingly, functional pathway enrichment on their associated genes identified the WNT signaling pathway and pluripotency network, etc., for cluster 1, embryonic development-related pathways to cluster 2, and gastrulation-related pathways to cluster 3 (Figure 2D). Notably, EpiLSCs presented greater chromatin openness both in cluster 1 compared with EpiLCs (Figure 2C; p < 2.2e–16, paired sample t test) and in cluster 2 compared with ESCs (Figure 2C; p < 2.2e–16, paired sample t test). In contrast, EpiLSCs displayed less chromatin accessibility in cluster 3 compared with EpiSCs (Figure 2C; p < 2.2e–16, paired sample t test). Moreover, we found that specific peaks at enhancer and/or transcription start sites (TSSs) were similarly enriched in ESCs and EpiLSCs for naive pluripotency genes such as *Klf4* and *Tdh* (Figure 2E), but EpiLSCs also displayed increased chromatin accessibility across enhancers for formative pluripotency marker genes, such as *Fgf5* and *Pou3f1* (Figure 2E). Specific peaks at enhancers of primed pluripotency marker genes such as *Lin28a* and *Krt8* were comparable in EpiLSCs and EpiLCs but lower than in EpiSCs (Figure 2E). These results further suggested that EpiLSCs harbored distinct chromatin features from EpiLCs, in line with the intermediate state of naive to primed pluripotency.

As chromatin priming often proceeds activation of developmentally regulated genes, we examined the active enhancer regions identified for ectoderm-, mesoderm-, and endoderm-specific genes at E7.5.^31^ We found that the ectodermal enhancers were more open in each cell type (Figure S2B) compared with those of mesoderm (Figure S2C) and endoderm (Figure S2D), confirming that somatic lineage-neutral PSCs are poised for ectoderm differentiation as a ‘‘default state.’’^31,32^ Specifically, EpiLSCs displayed significantly greater chromatin accessibility than ESCs (p < 2.2e–16, paired sample t test) and EpiSCs (p < 2.2e–16, paired sample t test) in the ectoderm-lineage enhancers (Figure S2B). A set of super bivalent genes is identified to prelude chromatin state reconfiguration essential for the transition from naive to primed pluripotency upon implantation.^32^ We found that TSS regions (i.e., ±5 kb) of these super bivalent genes were most accessible in EpiLCs, followed by EpiLSCs, ESCs, and EpiSCs (Figures 2F and 2G). Regarding competence for germline specification,^16^ we investigated the TSS openness of PGC-related genes.^14^ Notably, PGC-related genes *Hoxb1*, *Prdm1*, and *Prdm14* are also identified as super bivalent genes and showed higher peak intensity in EpiLSCs and EpiLCs (Figure 2G). In contrast, *Hoxb1* and *Prdm1* were closed in ESCs, and *Prdm14*, *Hoxb1*, and *Prdm1* were closed in EpiSCs (Figure 2G). Like EpiLCs, EpiLSCs also displayed stronger TSS openness in other PGC-related genes, including *Dnd1*, *Dppa3*, and *Sox15* (Figure 2G).

To further elucidate the effects of WNT/β-catenin signaling on EpiLSCs’ chromatin landscape, we next examined genes involved in canonical WNT pathways that have higher peak intensities in EpiLSCs than EpiSCs. We found that 46 genes including co-receptors *Lgr4*, *Lrp5*, and *Fzd4*; modulators *Dkk3* and *Sfrp*; and downstream targets *Ctnnd1*, *Rbpj*, *Mbd2*, *Hdac2*, and *Myc* were more enriched in EpiLSCs (Figure 2H). Interestingly, *Rbpj* is reported to prevent expression of naive pluripotency genes and facilitate the naive state exit.^33^ Overall, these results suggested that the chromatin landscape of EpiLSCs was reconfigured by WNT/β-catenin signaling toward a lineage-neutral formative state.

### EpiLSCs display transcriptional heterogeneity and recapitulate the pluripotency transition before gastrulation *in vivo*

**Figure 3.**
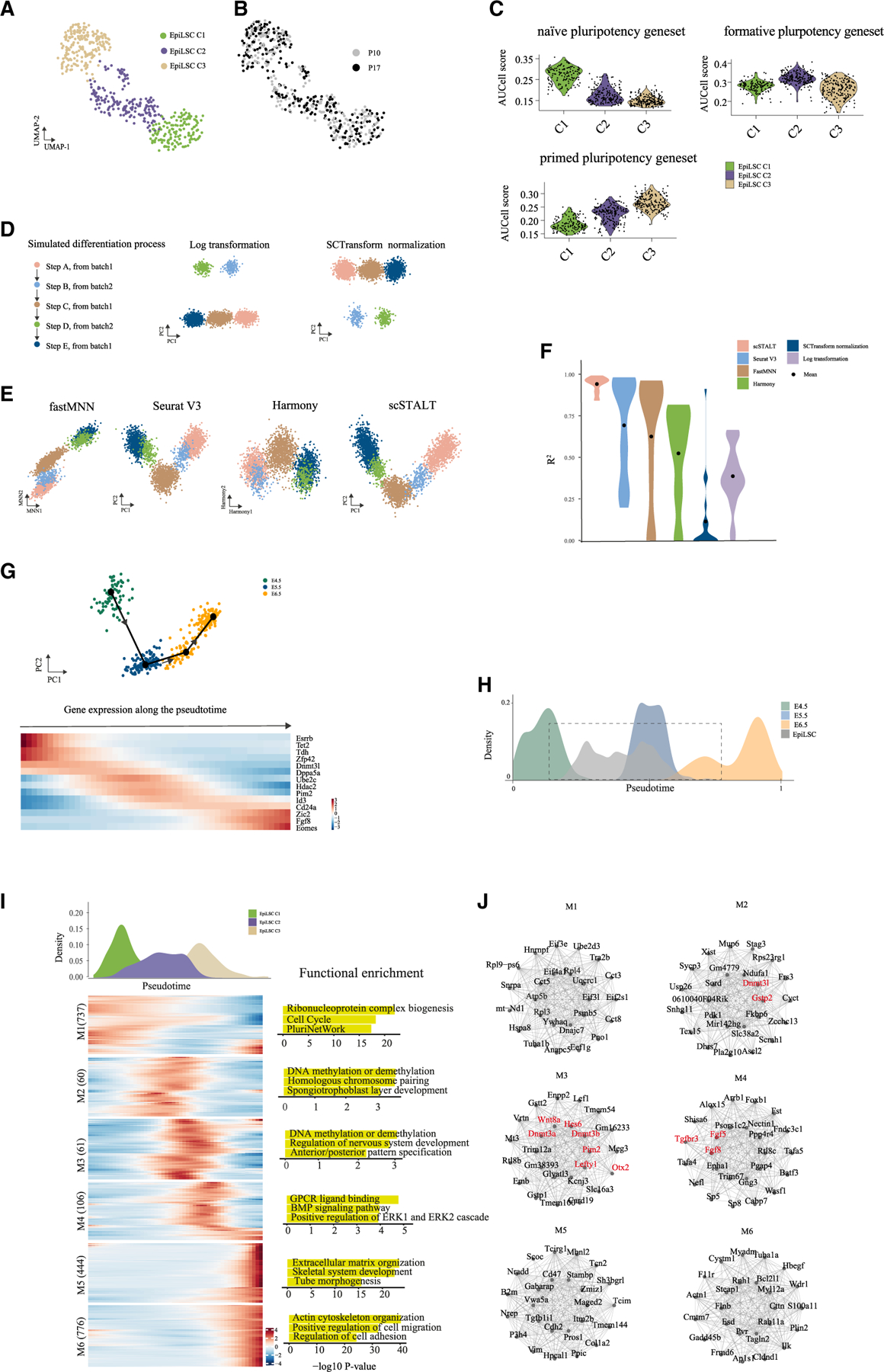
EpiLSCs display transcriptional heterogeneity of pluripotency and recapitulate the *in vivo* pluripotency transition (A and B) UMAP embedding and clustering of single-cell RNA-seq of EpiLSCs (A) and presented with color indicating the passage of the EpiLSCs (B). (C) Cell activities of the naive, formative, and primed gene sets of EpiLSCs. (D) Simulation of a differentiation process in the order of A to E with introduced batch effect (left). The visual embedding under log transformation (middle) and SCTransform nomalization (left). (E) Integration of the simulated datasets in (D) via fastMNN, Seurat v.3, Harmony, and scSTALT. (F) Benchmark of the pseudotime estimation of simulated datasets by R^2^ coefficient. (G) Pseudotime trajectory of E4.5–6.5 epiblast indicated with the arrow (top) and the dynamic genes shown in the heatmap (bottom).(H)The pseudotimes of E4.5, E5.5, and E6.5 were distributed in the range from 0 to 1. EpiLSCs were projected by scSTALT to the timescale to show their counterpart equivalent *in vivo* state. (I) The scWGCNA identified gene modules of EpiLSCs. M1–M6 stand for 6 distinctive gene modules, with gene numbers in each module following the module name in the brackets. The functional enrichment implications are shown with –log10 p values (hypergeometric test) on the right. (J) Network of the hub genes of the modules. See also Figure S3 and Tables S2 and S3.

To dissect the cellular composition of EpiLSCs, we performed single-cell Smart-seq3 RNA sequencing (RNA-seq) on 528 high-quality cells harvested from passages 10 and 17 (Figures S3A and S3B). We identified three clusters (C1–C3) using shared nearest neighbor (SNN) modularity optimization (Figure 3A) in which cells from two passages exhibited similar distributions (Figures 3B and S3C; chi-squared test, p = 0.21). Further examination of pluripotency gene set activities identified enrichment of a naive gene set in C1 and a formative and primed gene set in C2 and C3, respectively (Figure 3C). Notably, C1 cells also expressed formative pluripotency genes such as *Dnmt3l*, *Zic2*, and *Etv5*, whereas C2 cells sparsely expressed naive pluripotency genes and C3 cells presented formative gene expression in addition to primed gene expression (Figure S3D). These results suggested that EpiLSCs constituted a heterogeneous but stable population spanning the naive-to-primed pluripotency continuum.

To correlate EpiLSCs with the epiblast and published formative PSCs, we developed an integrative pseudotime estimation algorithm, scSTALT, that did not require mutual neighbors or shared anchors as by Seurat v.3^34^ and mutual nearest neighbor (MNN)^35^ and least resulted in overfitting. To test our method, we simulated a continuous differentiation process from steps A to E and introduced batch effects to A, C, and E as one batch and B and D as the other^36^ (Figure 3D). By log transformation or SCTransform^37^ normalization, clear separation of the two batches was seen in the PCA plot (Figure 3D). After integration with Seurat v.3^34^, FastMNN,^35^ and Harmony,^38^ the incurred overfitting was evident as indicated by the convergence between adjacent clusters (A/B, D/E) from the two batches (Figure 3E). In contrast, scSTALT accurately recovered the trajectory (Figure 3E). We repeated simulations 250 times with various combinations of signal-to-noise ratios and non-linear gene proportions. To benchmark the accuracy of the integration, we compared the R^2^ efficiency of the inferred with simulated cell stages and found that scSTALT outperformed the other methods with an average R^2^ of 93.8 (Figure 3F).

Next, using scSTALT, we integrated EpiLSCs with published single-cell transcriptomic data from mouse epiblast cells.^39^ Briefly, we first used Slingshot to generate a pseudotime trajectory of E4.5–6.5 epiblast cells (Figures 3G and S3E). Then, we estimated the dynamic gene expressions and obtained a kernel expression profile with 538 dynamic genes (Figure S3F; Table S2), including pluripotency-associated genes such as *Esrrb*, *Tet2*, *Tdh*, and *Zfp42* (Figure 3G). Using the kernel expression profile, we successfully aligned the developmental trajectory using single-cell RNA-seq of E4.5–6.5 epiblasts^31^ and bulk RNA-seq of E4.75 and E5 epiblasts^40^ (Figure S3G), which further confirmed that the kernel gene expression profile was robust for pseudotemporal ordering of cells. We then utilized the kernel expression profile for assigning EpiLSCs along the trajectory and found that EpiLSCs were mostly distributed between E5 and E6, greatly overlapping with E5.5 epiblast cells, suggesting capture of early post-implantation development (Figure 3H).

To determine the gene regulatory network in EpiLSCs, we used single-cell weighted gene co-expression network analysis (scWGCNA)^41^ on EpiLSCs and identified six major modules, M1–M6 (Figure 3I; Table S3). Interestingly, the M1–M6 modules showed distinct dynamic patterns along the pseudotime trajectory, indicating pluripotency transition dynamic networks (Figure 3I). Among the six modules, M1 was mostly expressed in the C1 cells with pluripotency-associated pathways (Figure 3I). M2 and M3 were highly expressed in C2 cells (Figure 3I). M2 expression preceded M3 slightly, but both contained genes enriched in DNA methylation or demethylation (Figure 3I), which plays an important role in naive pluripotency exit. M4 was mainly expressed in C3 cells and enriched in various signaling pathways. M5 and M6 were mostly enriched in some cells of C3 with gene functions likely involved in the gastrulation-related pathways such as tube morphogenesis, suggesting primed pluripotency states in line with the pseudotime trajectory analysis (Figure 3I). For each module, we further listed the top 25 hub genes showing the interaction networks (Figure 3J). Notably, several hub genes in M2, M3, and M4 were previously annotated as formative pluripotency genes such as *Dnmt3l*, *Otx2*, and *Fgf5* (Figure 3J, highlighted in red), which is consistent with the conclusion that formative pluripotency is captured in EpiLSCs.

### EpiLSCs harbor distinct molecular features compared with other formative PSCs

**Figure 4.**
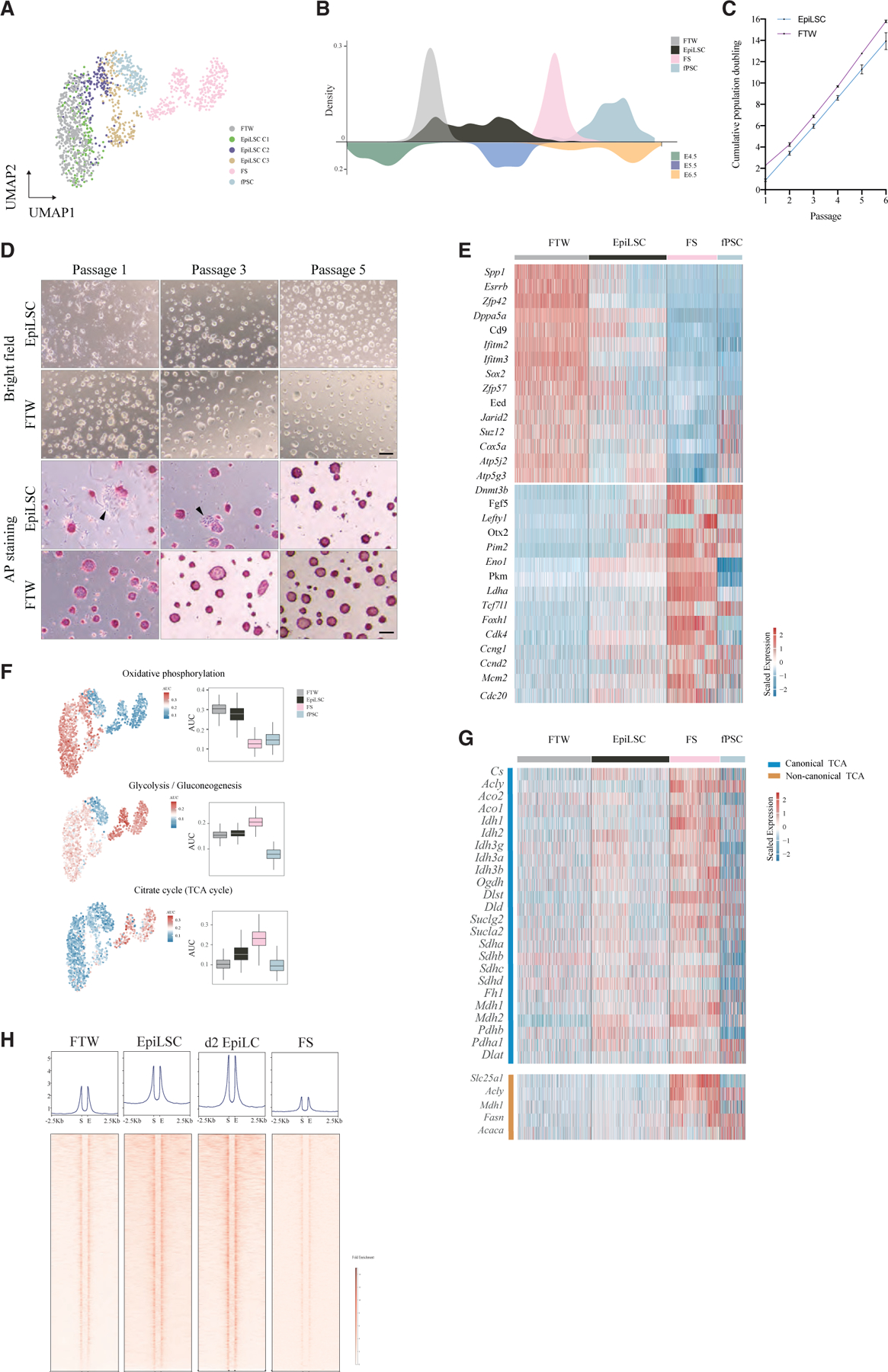
Comparison of EpiLSCs with other formative PSCs (A) UMAP embedding of EpiLSCs with FTW cells, FS cells, and fPSCs. (B) Aligning formative PSCs in (A) to the E4.5–6.5 epiblasts by scSTALT. (C) Cumulative population doubling of FTW cells and EpiLSCs during adaptation in 2i+LIF medium. (D) Morphology of FTW-ESCs and EpiLSCs during their adaptation in 2i+LIF medium shown by phase contrast (scale bar, 200 μm) and AP staining (scale bar, 100 μm) images. Arrowheads indicate differentiated colonies. (E) Heatmap showing the expressions of the selected consecutively downregulated genes (FTW cells > EpiLSCs > FS cells) and upregulated genes (FTW cells < EpiLSCs < FS cells). (F) Metabolic pathway transcriptional activity inferred by scMetabolism shown in the same UMAP as in (A) (left) and in bar plot (right). (G) Heatmap showing the gene expressions of canonical and non-canonical TCA cycle genes in FTW cells, EpiLSCs, FS cells, and fPSCs. (H) ATAC-seq openness of FTW cells, EpiLSCs, day 2 EpiLCs, and FS cells in PRC2 insulators showing by metagene profile plot (top) and heatmap (bottom). S and E stand for the starting and ending sites of the insulator region, respectively. 5 kb flanking regions were included. See also Figure S4 and Table S4.

FTW cells,^18^ FS cells,^19^ and fPSCs^20^ are recently established formative PSCs. We thereafter compared EpiLSCs with them. As there was no available FTW single-cell RNA-seq data, we first used Smart-seq3 to generate 500 high-quality FTW cells to be analyzed together with 338 FS cells^19^ and 168 fPSCs.^20^ We found that each cell type was clustered together and sequentially ordered as FTW cells, EpiLSCs, fPSCs, and FS cells (Figure 4A). The three clusters of EpiLSCs were distinguishable with C1 EpiLSCs intermingled with FTW cells while C3 was closer to fPSCs and FS cells (Figure 4A). To further explore the pluripotency states of FTW cells, FS cells, and fPSCs, we used scSTALT and found that FTW cells resembled the E4.5–5 epiblast, whereas FS cells and fPSCs were closely related to the E6.5 epiblast (Figure 4B). Interestingly, there was little overlap of each cell type except for FTW and C1 EpiLSCs. EpiLSCs filled the gap of formative pluripotency continuum between FTW and FS cells and mostly resembled the E5.5 epiblast together with high expression of E5.5-specific genes (Figures S4A–S4C), suggesting that EpiLSCs recapitulate unique formative pluripotency bridging FTW and FS cells. Of note, fPSCs bookend the formative continuum and express several primitive streak-specific genes as reported.^20^ In accordance with our finding that FTW cells and EpiLSCs represent the early phase of formative pluripotency, both could adapt in 2i+LIF culture with continuous population doublings of 2.6 ± 0.4 and 2.3 ± 0.7 each passage, respectively (Figure 4C). We also observed round and dome-shaped colonies with AP^+^ staining (Figure 4D). Different from FTW cells, however, we noticed that EpiLSCs contained some differentiated colonies (i.e., stained negative for AP) in the first 3 passages, which were not seen from passage 5 (Figure 4D, arrowheads). We next investigated the distinctive gene expression pattern underlying the different pluripotency states by comparing EpiLSCs versus FTW cells and EpiLSCs versus FS cells, respectively. We found that 225 and 305 genes showed gradual downregulation and upregulation, respectively, from FTW cells and EpiLSC to FS cells (Figure S4D; Table S4). Among the downregulated genes, we noticed several naive pluripotency markers such as *Spp1*, *Esrrb*, *Zfp42*, and *Dppa5* (Figure 4E). In contrast, several formative pluripotency markers such as *Dnmt3b*, *Fgf5*, *Lefty1*, *Otx2*, and *Pim2* were among the upregulated genes (Figure 4E).

We also observed downregulation of oxidative phosphorylation complexes genes *Cox5a*, *Atp5j2*, and *Atp5g3* and upregulation of glycolysis associated genes *Pim2*, *Eno1*, *Pkm*, and *Ldha* following formative pluripotency progression (Figure 4E), in line with the Seahorse analysis (Figures 1C and 1D). We further calculated the metabolism pathway enrichment on each cell type using scMetabolism^42^ and found comparable metabolic activities of oxidative phosphorylation and glycolysis between FTW cells and EpiLSCs (Figure 4F). In contrast, FS cells showed preference for glycolysis (Figure 4F). TCA cycle activity can regulate pluripotency by influencing chromatin modifications and DNA methylation.^43^ Notably, TCA cycle activity was clearly increased from FTW cells to FS cells (Figure 4F). In addition to TCA cycle genes, FS cells simultaneously expressed increased levels of non-canonical TCA cycle genes (Figure 4G) that shuttle citrate from mitochondria into cytosol and carry out proton-generating biochemical reactions in the cytosol.^44^ The non-canonical TCA cycle is reported to accompany the switching of pluripotency states in ESCs.^44^ Unexpectedly, fPSCs showed relatively low metabolic activity in all three pathways examined (Figure 4F), likely due to their low transcriptional activities and/or sequencing depth (Figure S4E).

Furthermore, polycomb-repressive complex 2 (PRC2) subunit genes *Eed* and *Suzl2* as well as co-factor *Jarid2* were gradually downregulated (Figure 4E). FTW cells showed the highest expression of PRC2 subunit genes, in line with the previous findings that PRC2 is required to maintain naive pluripotency in a hypomethylated state, with open chromatin shielding them from differentiation,^45^ agreeing with H3K27me3 enrichment between E4.5 and E5.5.^32^ Using 5,825 identified PRC2-silenced regions in ESCs,^46^ we next examined their chromatin accessibility in FTW cells, EpiLSCs, and FS cells.^19^ We found that the decreasing expression of PRC2 subunits in EpiLSCs (Figure 4E) were accompanied by enhanced chromatin openness at the PRC2-silenced regions compared with FTW cells (Figure 4H). These regions were even more accessible in day 2 EpiLCs (Figure 4H). However, FS cells showed lower chromatin accessibility in these regions (Figure 4H), which could be due to inhibition of WNT/β-catenin signaling and/or increased methylation as a result of high levels of *Dnmt3a* expression (Figure 4E). Taken together, these results showed that EpiLSCs represent a unique intermediate formative state between FTW and FS cells. In addition, we found that different formative PSCs harbored distinctive metabolism states and epigenetic regulators.

### WNT/β-catenin signaling sustains a metastable formative state in EpiLSCs

**Figure 5.**
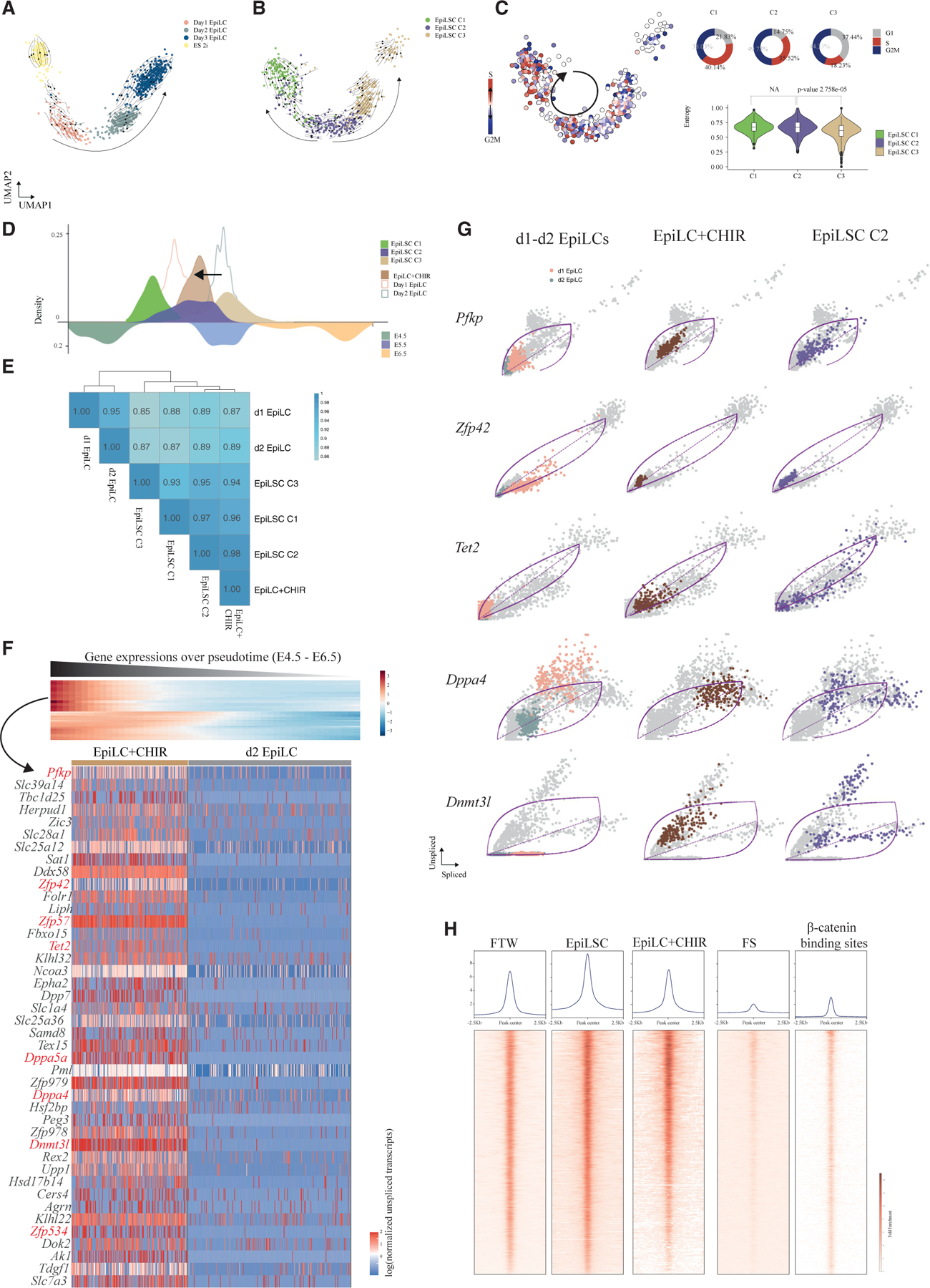
WNT/β-catenin signaling sustains dynamic cell states in EpiLSCs to maintain the formative pluripotency (A and B) Velocities of ESCs and day 1–3 EpiLCs (A) and C1–C3 EpiLSCs (B). (C) Cell-cycle score presented in the same scSTALT embedding as in (B) (left). Cell-cycle phase distribution and cell entropies were presented cluster-wise in the pie chart (right). (D) Pseudotime projection of EpiLCs+CHIR compared with day 1–2 EpiLCs and EpiLSCs. (E) Spearman correlation matrix of the expression profile of day 1–2 EpiLCs, C1–C3 EpiLSCs, and EpiLCs+CHIR. (F) CHIR increases the expression potential of the 42 driver genes. The inferred driver genes were expressed in the early phase during E4.5–6.5 trajectory (top), and their unspliced/spliced ratios in EpiLC+CHIR are higher than those in day 2 EpiLCs (bottom). (G) Velocities of the selected genes in day 1–2 EpiLCs, EpiLCs+CHIR, and C2 EpiLSCs. (H) ATAC-seq openness of FTW cells, EpiLSCs, day 2 EpiLCs+CHIR, and FS cells in the β-catenin binding sites shown by metagene profile plot (top) and heatmap (bottom). 5 kb flanking regions were included. See also Figure S5.

To elucidate the effects of WNT/β-catenin signaling on sustaining EpiLSCs, we first performed differentially expressed gene analysis comparing EpiLSCs with day 2 EpiLCs. We found that C2 EpiLSCs were most similar to day 2 EpiLCs with the fewest differentially expressed genes. In addition, C1 EpiLSCs upregulated naive pluripotency genes, whereas C3 EpiLSCs upregulated primed pluripotency genes compared with day 2 EpiLCs (Figure S5A; Table S5). By scSTALT, we further confirmed that C2 EpiLSCs were most similar to day 2 EpiLCs at the global gene expression level (Figure S5B). Next, we compared the cellular state transitions from day 1 to 3 EpiLCs^29^ to EpiLSCs using scVelo.^47^ To compare in the same vector field, we firstly used scSTALT to integrate the day 1–3 EpiLCs and EpiLSCs into a shared embedding (Figure S5C). Then, we performed scVelo on day 1–3 EpiLCs (Figure 5A) and EpiLSCs (Figure 5B), respectively. For EpiLCs, scVelo revealed the directional differentiation trajectory consistent with the sampling time (Figure 5A). On the other hand, the three different states of EpiLSCs orderly aligned along the trajectory (Figure 5B). Interestingly, in addition to the directional differentiation of EpiLSCs from C1 to C3 cells, we also observed a bifurcation in C2 where some cells displayed the reversed trajectory to C1 (Figure 5B), suggesting that WNT/β-catenin signaling counteracted the directional differentiation. This was further supported by the observation that passage 10 and 17 EpiLSCs were distributed similarly along the trajectory (Figure S5C). Moreover, compared with C3 EpiLSCs, C1 and C2 EpiLSCs had a higher percentage of S and G2/M phases and a lower percentage of G1 phase (Figure 5C). We also estimated the cell entropy as a robust proxy for pluripotency and cell differentiation potency.^48^ The entropy was higher in C1 and C2 EpiLSCs compared with that in C3 EpiLSCs (Figure 5C). The velocity together with cell-cycle and entropy results conclude that C1 and C2 cells constitute the cycling pluripotent population that are readily responsive to signaling cues, whereas C3 is more differentiated. We calculated the distribution of passage 10 and 17 EpiLSCs regarding their cycling and non-cycling populations. The result showed that they have comparable proportions of active cycling population, suggesting stability of EpiLSCs across passages (Figure S5D).

To further reveal the molecular mechanism by which WNT/β-catenin signaling sustained the formative pluripotency and counteracted differentiation, we acquired Smart-seq3 single-cell RNA-seq of 198 cells collected from day 2 EpiLCs cultured with CHIR09921 supplementation (named EpiLCs+CHIR). First, we ordered EpiLCs+CHIR cells together with day 1–2 EpiLCs and EpiLSCs by scSTALT (Figure 5D). Interestingly, we found that compared with day 2 EpiLCs, EpiLCs+CHIR cells were shifted toward an earlier developmental time closer to day 1 EpiLCs and corresponded to C2 EpiLSCs (Figure 5D). We further confirmed that EpiLCs+CHIR cells were mostly similar to EpiLSCs, especially C2 EpiLSCs (Figures 5E and S5E), suggesting that CHIR09921 supplementation thwarted the differentiation and enabled EpiLC self-renewal in the formative pluripotency state.

As RNA velocity reveals transcriptional dynamics, we identified upregulation latencies in EpiLCs+CHIR cells compared with day 2 EpiLCs, followed by overlapping with downregulated genes from E4.5 to E6.5 *in vivo* epiblast cells. In total, we found 42 genes as potential downstream targets of WNT/β-catenin signaling to counteract EpiLC differentiation (Figures 5F and S5F). Among these genes, zinc-finger protein genes *Zfp42*, *Zfp57*, and *Zfp534* and DNA methylation regulators *Tet2* and *Dnmt3l*, as well as *Dppa5a* and *Dppa4*, are known to regulate pluripotency maintenance^49,50^ (Figure 5F, highlighted in red). Interestingly, we also identified *Pfkp*, which may account for different metabolic features in EpiLSCs and EpiSCs (Figure 5F). Since C2 is the branching cluster in EpiLSCs, we compared gene-wise splicing kinetics in day 1–2 EpiLCs, EpiLCs+CHIR cells, and C2 EpiLSCs for these 42 genes (Figures 5G and S5G). The dynamics for each gene were modeled by scVelo with a dashed line indicating a steady state (neutral unspliced/spliced ratio) that demarcates the upregulation and downregulation as above or below the line, respectively. In day 1–2 EpiLCs, most genes showed low ratio of unspliced/spliced transcripts at the zero point (i.e., low in both splice and unspliced transcripts), indicating inactive transcription. And this was found to be more obvious in day 2 EpiLCs than day 1 EpiLCs (Figure 5G). In EpiLCs+CHIR cells, these genes became activated and upregulated toward activation state in C2 EpiLSCs (Figure 5G) and became more active in C1 EpiLSCs (Figure S5G).

To further study the effects of WNT/β-catenin signaling on formative pluripotency maintenance, we examined the chromatin accessibility of EpiLCs+CHIR and EpiLSC on the β-catenin binding regions identified by chromatin immunoprecipitation (ChIP)-seq in ESCs.^51^ EpiLCs+CHIR cells had increased accessibility on these β-catenin binding regions, which were also open in EpiLSCs and FTW cells (Figure 5H). In contrast, FS cells lacked corresponding chromatin openness as in accordance with inhibition of WNT/β-catenin signaling (Figure 5H). Further examination revealed that FTW ATAC-seq captured 8,087 peaks overlapping with β-catenin binding sites; EpiLSCs and EpiLCs+CHIR had 6,752 and 6,447 peaks, respectively, whereas FS cells only had 994 peaks (Figure S5H). Motif analysis of these overlapping peaks showed top enriched TFs OSTN, *Lef1*, and *Tcf3* (*Tcf7l1*) for the active binding sites in FTW cells, EpiLSCs, and EpiLCs+CHIR, indicating that β-catenin mainly functions through derepressing and activating pluripotency genes in these cells (Figure S5I). In contrast, the overlapping peaks in FS cells enriched in OSTN, *Brn1*, and *Sox15* (Figure S5J). Taken together, these results showed that WNT/β-catenin signaling activated pluripotency genes and sustained formative pluripotency programs in cultured EpiLSCs.

### EpiLSCs display direct competence for germline induction *in vitro*

**Figure 6.**
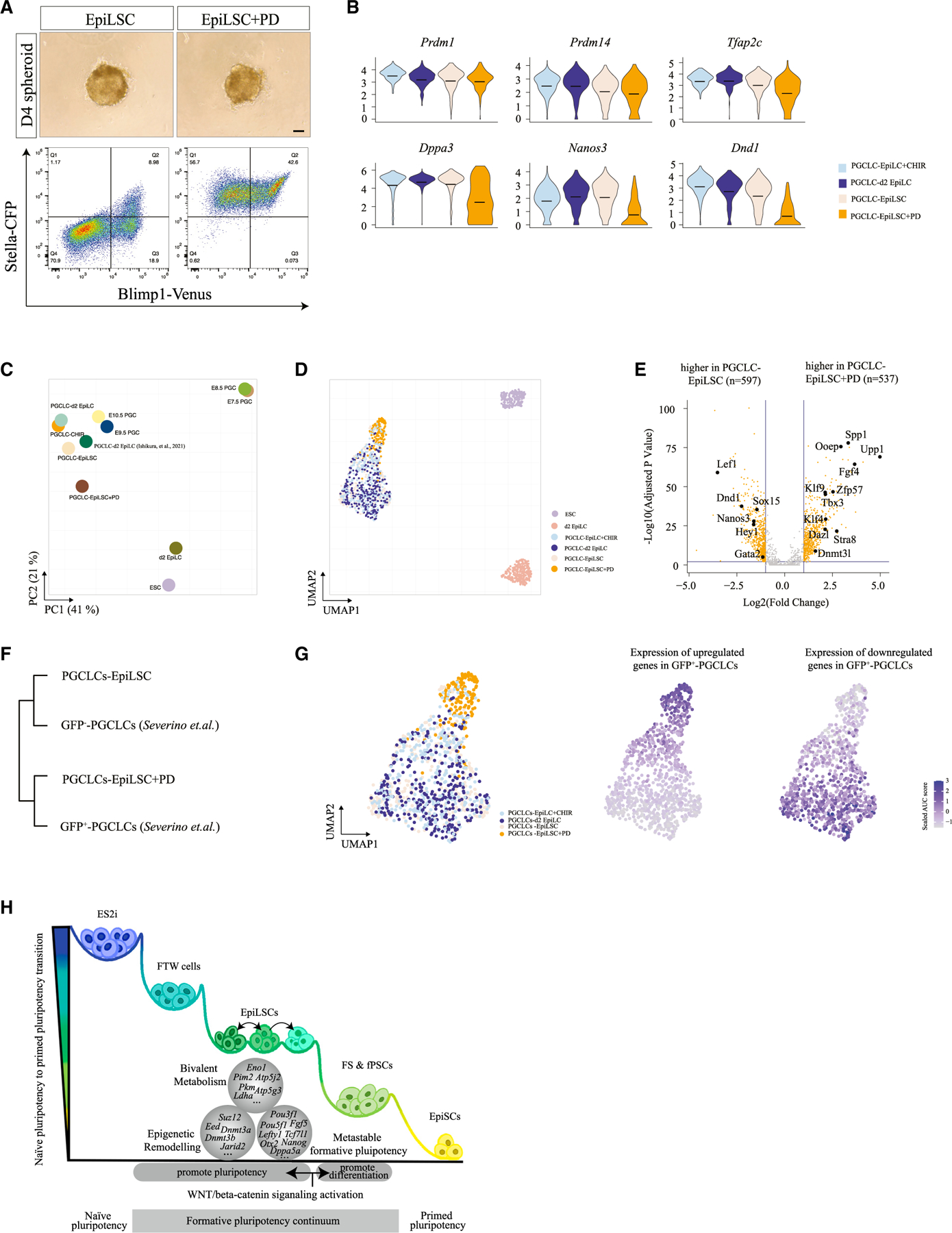
Direct competence of EpiLSCs for germline induction *in vitro* (A) The morphology of spheroids at day 4 of PGCLC induction with/without PD supplementation (top). Scale bar, 100 μm. Fluorescence-activated cell sorting (FACS) of dissociated day 4 spheroids with Stella-CFP and Blimp1-Venus reporters showing the detection of PGCLCs (bottom). (B) Expressions of PGC marker genes in PGCLC-day 2 EpiLCs, PGCLC-EpiLCs+CHIR, PGCLC-EpiLSCs, and PGCLC-EpiLSCs+PD. (C) PCA plot of PGCLCs, PGCs, ESCs, and day 2 EpiLCs. PGCLC-day 2 EpiLCs, PGCLC-EpiLCs+CHIR, PGCLC-EpiLSCs, PGCLC-EpiLSCs+PD, E7.5 PGCs, E8.5 PGCs, ESCs, and day 2 EpiLCs. (D) UMAP embedding of PGCLC-day 2 EpiLC, PGCLC-EpiLC+CHIR, PGCLC-EpiLSC, PGCLC-EpiLSC+PD, ESC, and day 2 EpiLC single-cell RNA-seq data. (E) Volcano plot showing the differentially expressed genes between PGCLC-EpiLSCs and PGCLC-EpiLSCs+PD. Orange dots means log2(fold change) >1 and adjusted p value <0.05. (F) Dendrogram clustering showing PGCLC-EpiLSCs with GFP^−^-PGCLCs and PGCLC-EpiLSCs+PD with GFP^+^-PGCLCs.(G)AUCell scoring of the gene sets in the UMAP embedding. (H) Illustration showing the formative pluripotency continuum captured by EpiLSCs, FTW cells, FS cells, and fPSCs. See also Figure S6 and Table S6.

In mice, one key distinguishing feature of formative pluripotency is the direct competence for germline induction.^16^ We therefore examined PGCLC specification from EpiLSCs starting from spheroid formation in response to the cytokine cocktail. We examined PGCLC induction efficiency in day 4 spheroids according to double positivity of CFP and Venus.^22,23^ As the FGF receptor (FGFR) inhibitor PD greatly increases PGCLC induction efficiency,^18^ we compared PGCLC yield with and without PD treatment. Our results showed that EpiLSCs generated 5%–10% and 35%–50% BV^+^SC^+^ cells with and without PD supplementation, respectively (Figure 6A). We also derived another EpiLSC line using Oct4-DE-EGFP mESCs,^52^ which showed similar morphology and cellular characteristics as BVSC-EpiLSCs (Figure S6A). Interestingly, the PGCLC induction efficiency is 50%–60% with PD treatment (Figures S6B–S6D), which is in line with the previous reports showing that differentiation efficiency varies among formative pluripotent cell lines.^18,19^

As little is known about how PD enhances PGCLC production, we compared the molecular identity of PGCLCs induced from EpiLSCs with and without PD. We sorted the BV^+^SC^+^ population at day 4 and performed Smart-seq3 single-cell RNA-seq. We generated 304, 321, and 161 high-quality day 4 PGCLC libraries induced from EpiLCs+CHIR, EpiLSCs, and EpiLSCs+PD, respectively, in addition to 303 day 4 PGCLCs induced from day 2 EpiLCs as control. First, we confirmed the PGC-like identity of these cells by examining the expression of PGC-specific marker genes, including *Prdm1*, *Prdm14*, *Tfap2c*, *Dppa3*, *Nanos3*, and *Dnd1* (Figure 6B). Interestingly, the PGCLCs induced with PD supplementation (i.e., EpiLSCs+PD) showed lower expressions of *Dnd1* and *Nanos3* (Figure 6B). To further explore the identity of these day 4 PGCLCs, we systematically compared them with previously published bulk and single-cell data including day 4 PGCLCs from day 2 EpiLCs,^53^ E7.5–10.5 PGCs,^54–56^ day 2 EpiLCs, and ESCs.^29^ As shown by principal-component analysis (PCA), all day 4 PGCLCs from four conditions were closely clustered in relation to previously published day 4 PGCLCs and E9.5 and 10.5 PGCs but were distantly separated from ESCs, day 2 EpiLCs, and E7.5 and E8.5 PGCs (Figure 6C). Interestingly, PGCLCs derived from EpiLSCs+PD were separated from other conditions. To further confirm this, we projected single-cell data into uniform manifold approximation and projection (UMAP) embedding and found that the PGCLCs induced from day 2 EpiLCs, EpiLCs+CHIR, and EpiLSCs intermingled together but less so with PGCLCs from EpiLSCs+PD (Figure 6D). To un-cover the underlying differences between the PGCLCs generated from EpiLSCs with/without PD supplementation, we performed differential gene expression analyses. We obtained 537 upregulated and 597 downregulated genes in the PD-treated day 4 PGCLCs (Figure 6E; Table S5). We noticed that several upregulated genes were related to naive pluripotency maintenance such as *Klf4*, *Tbx3*, *Zpf57*, and *Spp1* (Figure 6E). Notably, some upregulated genes in PGCLCs derived from EpiLSCs+PD were associated with meiotic cell cycle, such as *Dazl*, *Dnmt3l*, and *Stra8* (Figure 6E). Interestingly, a recent study revealed that female day 5 PGCLCs with two activated X chromosomes (GFP^+^-PGCLCs) have also upregulated the aforementioned genes compared with those with one activated X chromosome (GFP^−^-PGCLCs).^57^ We thus compared our day 4 PGCLCs with/without PD with these female PGCLCs. As our PGCLCs were male cells, we used autosomal differentially expressed genes and found that PGCLCs derived from EpiLSCs with PD were clustered with GFP^+^-PGCLCs, whereas PGCLCs without PD treatment were clustered with GFP^−^-PGCLCs (Figure 6F). Next, we applied AUCell^58^ and found that PGCLCs with PD treatment highly expressed genes upregulated in GFP^+^-PGCLCs, while PGCLCs without PD treatment showed a higher expression of genes upregulated in GFP^−^-PGCLCs (Figure 6G). Therefore, PD treatment not only enhanced PGCLC induction efficacy but also potentially contributed to their maturation. It would be interesting to examine if PD-treated PGCLCs are better suited for *in vitro* gametogenesis in the future.

## DISCUSSION

Mouse naive ESCs and primed EpiSCs recapitulate two opposite ends of the pluripotency spectrum. The dismantling of naive pluripotency by inductive signaling such as activin A and bFGF occurs in an orderly manner that first activates transcriptional programs establishing formative states characteristic of early post-implantation epiblast cells. This dynamic process was initially modeled by transitory EpiLCs showing a short window of competence for both soma and germline specification.^14^ Our current study, together with recently published formative stem cells, can stabilize these transient cell states by manipulating WNT/β-catenin signaling, resulting in distinct molecular properties as summarized in Figure 6H.

### Formative pluripotency represents an intermediate spectrum from E5 to E6.5 epiblast

Although EpiSCs can be derived from E5–7 epiblasts, they invariably converge on molecular traits closely related to E7 epiblast.^5,59^ Formative pluripotency is proposed to represent an intermediate spectrum recapitulating E5 to E6.5.^16^ It has been shown that three recently reported formative PSCs, i.e., FTW cells,^18^ FS cells,^19^ and fPSCs,^20^ are different in global gene expression as well as their *in vivo* counterparts. Among these, FTW is closest to naive pluripotency and resembles ~E5 epiblast, which reflects their origin from E3.5 blastocyst and culture condition on feeders supplying LIF.^18^ Consistently, isolated E5.25–6.25 epiblasts either differentiated or died before passaging when using FTW culture condition.^18^ On the other hand, FS cells are derived from E5.5 epiblast but cultured for 5–6 days as explants before stabilization, which explains their resemblance to E6–6.5 epiblast. In comparison, a great majority of EpiLSCs harbor the molecular features bridging FTW and FS cells and resemble E5–6 epiblast. EpiLSCs and FTW cells shared the same culture parameters except for feeders. As a result, EpiLSCs appear metastable in morphology and diversified in molecular features, which is reminiscent of mouse ESCs grown in serum+LIF condition that resulted in heterogeneous and dynamic population of naive- and primed-like cells.^60^ Interestingly, EpiLSCs could be stably maintained for at least 20 passages. In a metastable system, cells do not have a rigidly fixed identity but instead can transit between co-existing attracting states.^61^ Molecular noise can trigger stochastic transitions between co-existing attractor states.^62^ As EpiLSCs are exposed to signaling milieu with counteracting factors promoting both primed (activin A and bFGF) and naive (WNT/β-catenin signaling activator) pluripotency, the environmental noise is greater than that of FTW cells, whose LIF signaling provided by feeders contribute to locking the cell state. As E5–6 embryos undergo extensive morphogenesis including anterior-posterior patterning, the epiblast exhibits great cellular heterogeneity as previously shown.^62^ This *in vivo* E5–6.5 epiblast expression profile could be approximated by transient EpiLCs *in vitro* and is lost upon stabilization in specific formative conditions including FTW cell, FS cell, and fPSC cultures. Interestingly, WNT/β-catenin signaling perpetuates EpiLC properties and stably maintains a metastable formative pluripotency population *in vitro*.

### Context-dependent function of WNT/β-catenin signaling when establishing formative pluripotency

Canonical WNT/β-catenin signaling supports naive mouse ESCs self-renewal and promotes EpiSC differentiation depending on the intertwined gene regulatory networks at play.^51^ TCF3 and β-catenin interact with the *Oct4-Sox2* complex to stabilize the naive pluripotency program but can also activate MEK/ERK pathways to induce lineage differentiation in primed pluripotency. Therefore, for FTW cells and EpiLSCs, when the naive pluripotency program is not completely dismantled, activation of WNT/β-catenin signaling pathway serves as a driving force to counteract differentiation caused by the supplementation of activin A and bFGF. For FS cells and fPSCs, when the naive pluripotency regulatory network is fully decommissioned, WNT/β-catenin signaling drives differentiation and necessitates inhibition to retain formative pluripotency. Previously, EpiSCs were also shown to compose of metastable, dynamic subpopulations in which OCT4^+^ and OCT4^−^ cells are interconvertible *in vitro*.^63^ Although OCT4^+^ EpiSCs represent a minor fraction, these cells resemble the early epiblast and can readily contribute to chimeras.^63^ According to our current understanding, this subpopulation may represent formative pluripotency.^63^ Moreover, inhibition of the WNT/β-catenin signaling pathway shifts the characteristics of EpiSCs toward anterior epiblast potentiating neuroectodermal fate.^19^ This suggests that once cells have committed onward, they are reconfigured and deprived of the lineage-neutral property. Therefore, FS cells need to be established by inhibition of the WNT/β-catenin of the E5.5 epiblast that is not fully primed.

Departure from naive pluripotency requires dissolution of multilayered naive pluripotency network followed by the installation of an alternative new gene regulatory network. Removal of WNT/β-catenin signaling releases TCF3’s repression on key naive TFs *Esrrb*, *Tfcp2l1*, *Nanog*, and *Klf4*, and MAPK/ERK signaling pathway activation by activin A and FGF signaling allows for the relocation of *Etv4* and *Etv5* to associate with the formative pluripotency regulatory network, which is essential to exit from the naive state. When activating WNT/β-catenin signaling in EpiLSC culture like in naive ESCs, TCF3’s repression on naive gene regulatory circuit is alleviated. As cultured for FTW cells, together with LIF signaling supplied by feeders, the naive pluripotency gene network is further strengthened. Therefore, different from previous formative culture systems that stabilize cells in one molecular ‘‘attractor’’ state, we introduce a counteracting balance force that results in fluctuations in gene and protein expression levels driving transitions between co-existing attractors but still ensures robust formative pluripotency properties at the population level. The advantage of such a system would be that cells are not locked in one state and are more responsive to external signaling. This also provides a unique platform mimicking the early post-implantation period for studying mechanisms underlying developmental decisions and transitions, as well as dissecting the progression of pluripotency to germline specification *in vivo*.

### PD treatment enhances germline specification efficiency and maturation

The PGCLC culture system serves as the starting point for *in vitro* reconstitution of gametogenesis.^64^ The differentiation efficiency is generally 5%–10%.^19,22,65^ The low efficiency imposes the technical challenge to acquire a large number of cells for downstream applications such as chemical screening, etc. Although PD was recently introduced to enhance mouse PGCLC induction efficiency,^18^ little is known about its global effects. We found that PD facilitates PGCLC maturation via upregulating meiotic-related genes. In early mammalian germline development, female X chromosome reactivation is a prominent epigenetic reprogramming event^66^ during their migration to the gonads.^67,68^ Recently, it has been shown that heterogeneity of X chromosome inactivation in the EpiLC stage resulted in two subpopulations of derivative PGCLCs.^57^ Interestingly, PGCLCs with two activated X chromosomes displayed higher expression of meiotic cycle genes such as *Dnmt3l*, Dazl, and *Stra8* as well as LIF response genes such as *Zfp42*, *Spp1*, and *Fgf4*,^57^ which is similar to male PGCLCs derived from PD treatment as shown here. These findings suggest future studies to determine if these PGCLCs are more competent for *in vitro* gametogenesis.

In summary, we showed that WNT/β-catenin signaling activation can sustain the metastable EpiLSC with formative pluripotency by balancing signals that promote both naive and primed pluripotency. It becomes increasingly clear that formative pluripotency is not a singular state but rather a broad spectrum between naive and primed pluripotency, and our method, scSTALT, helps to infer accurately the *in vivo* counterpart of the formative PSCs. Importantly, EpiLSCs recapitulate a unique developmental window *in vivo* with comparable heterogeneity and could be useful for future application to study the critical period of early post-implantation development. Meanwhile, our method, scSTALT, is a trajectory reference-query-based integration method that is capable of performing integration on non-representative differentiation datasets.

### Limitations of the study

Our culture condition without feeders renders metastable properties to EpiLSCs. It remains to be determined if EpiLSCs can maintain dynamic cellular states in the long term, although it is generally a rule of thumb to use low-passage stem cells for downstream application. EpiLSCs are generated by transforming ESCs with signaling factors instead of deriving them from early mouse embryos like other formative PSCs. It may not be equally efficient for chimera formation, although a previous study confirmed the possibility.^17^

## STAR★METHODS

### RESOURCE AVAILABILITY

#### Lead contact

Further information and requests for resources and reagents should be directed to and will be fulfilled by the lead contact, Qiaolin Deng (qiaolin.deng@ki.se).

#### Materials availability

This study did not generate new unique reagents. Reagent generated in this study will be made available on request, but we may require a payment and/or a completed Materials Transfer Agreement if there is potential for commercial application.

#### Data and code availability

- Raw ATAC-Seq and single-cell RNAseq data have been deposited in the sequence read archive and are publicly available as of the date of publication. Accession numbers are listed in the key resources table.
- All original codes for reproducing the results are deposited in GitHub and are publicly available as of the date of publication. The method scSTALT has been implemented in R package available in GitHub and is available as of the date of publication. DOIs are listed in the key resources table.
- Any additional information required to reanalyze the data reported in this paper is available from the lead contact upon request.

### EXPERIMENTAL MODEL AND STUDY PARTICIPANT DETAILS

#### Cell lines

BVSC mESCs^22,23^ were used in this study to generate EpiLSC, FTW-ESC and EpiSC. EpiLSCs were also generated from Oct4-DE-EGFP mESCs.^52^ ESCs were culture in N2B27 medium supplemented with 3 μM CHIR99021, 0.4 μM PD0325901 and 1000 U/mL LIF on gelatin-coated plates. EpiLSCs were generated from BVSC ESCs by continual culture in N2B27 medium supplemented with 20 ng/mL Activin A, 12 ng/mL bFGF, 3 μM CHIR99021 and 1% KSR on gelatin-coated plates. FTW-ESC were generated from BDF1-2 ESC by continual culture in N2B27 medium supplemented with 10 ng/mL Activin A, 10 ng/mL bFGF and 3 μM CHIR99021 on MEF-coated plates.^18^ EpiSC were generated from BDF1-2 ESC by continual culture in N2B27 medium supplemented with 20 ng/mL Activin A, 12 ng/mL bFGF and 1% KSR on fibronectin-coated plates. For the reversion of EpiLSC and FTW-ESC, the cells were seeded on gelatin-coated plates and cultured in 2i + LIF medium (N2B27 medium supplemented with 3 μM CHIR99021, 0.4 μM PD0325901 & 1000 U/mL LIF). Population doubling was calculated based on the formula ‘‘x = log(N2/N1)/log(2)’’ where N1 is the number of seeded cells and N2 is the number of harvested cells. The cumulative population doubling was calculated by adding population doubling of each passage to that of the previous passage. All cell lines were cultured with the supplementation of 1X penicillin-streptomycin, in a 37°C 5% CO_2_ humidified incubator. All experiments were carried out using cells cultured within 10–30 passages.

### METHOD DETAILS

#### PGCLC differentiation

PGCLC induction was performed following an established protocol.^80^ Briefly, Day 2 EpiLC were generated by culturing ESCs in N2B27 medium supplemented with 20 ng/mL Activin A, 12 ng/mL bFGF and 1% KSR on fibronectin-coated plates for 40–48 h, with one medium change 24 h after seeding. Then, viable cells were seeded in non-adherent 96-well round bottom plates at 2000 cells/well in 100 μL of GK15 medium (containing 15% KSR, 1X NEAA, 1 mM sodium pyruvate, 2 mM L-glutamine, 0.1 mM β-mercaptoethanol and 1X penicillin-streptomycin in GMEM) supplemented with 500 ng/mL BMP4, 500 ng/mL BMP8a, 100 ng/mL SCF, 50 ng/mL EGF, 1000 U/mL LIF and with/without 1 μM PD173074. The cells were cultured in a 37°C5% CO_2_ humidified incubator for 4 days.

#### Gastruloid differentiation

Gastruloid formation assay was performed following an established protocol.^81^ Briefly, viable cells were seeded in non-adherent 96-well round bottom plates at 300 or 800 cells/well in 40 μL of N2B27 medium and were cultured in a 37°C 5% CO_2_ humidified incubator for 2 days. At 48 h, 150 μL of N2B27 medium supplemented with/without 3 μM CHIR99021 was added to each well and further cultured for 1 day. At 72 h, 150 μL of medium was removed from each well and replaced with 150 μL of fresh N2B27 medium and further cultured for 1 day. This process was repeated at 96 h and the gastruloids were harvested at 120 h.

#### Somatic lineage differentiation

For mesoderm induction, EpiLSC were cultured in N2B27 medium supplemented with 20 ng/mL Activin A and 3 μM CHIR99021 on fibronectin-coated plates for three days with daily medium change. For endoderm induction, EpiLSC were cultured in N2B27 medium supplemented with 20 ng/mL Activin A and 3 μM CHIR99021 on fibronectin-coated plates for 24 h. Then the cells were cultured with N2B27 medium supplemented with 20 ng/mL Activin A only for the subsequent two days. For neural induction, EpiLSC were cultured in N2B27 medium on gelatin-coated plates for three days with daily medium change. All cell culture was performed in a 37°C 5% CO2 humidified incubator.

#### Alkaline phosphatase (AP) staining

Alkaline phosphatase staining was performed using AP detection kit (Sigma-Aldrich) following manufacturer’s instructions. Briefly, culture cells were fixed with 4% paraformaldehyde/PBS for 2 min at room temperature. The cells were rinsed with 1X PBS followed by 1X rinse buffer (containing 20 mM Tris-HCl (pH 7.4), 0.15 M NaCl & 0.05% Tween 20). Then, the cells were incubated with staining solution (containing fast red violet, Naphthol AS-BI phosphate & water at 2:1:1 ratio) for 15 min at room temperature. Then, the cells were rinsed with 1X rinse buffer and kept submerged in 1X PBS. Bright field images were taken using Evos XL core imaging system (Invitrogen).

#### Mitochondrial staining

Mitochondrial staining was performed as previously described.^82^ Briefly, cells were cultured on gelatin coated glass coverslip (0.16–0.19 mm thickness) in a 37°C 5% CO2 humidified incubator overnight. On the next day, the cells were incubated with fresh culture media containing 400 nM MitoTracker Red CMXRos (Invitrogen) for 30 min in the incubator. Then the media were replaced twice with fresh media and returned to the incubator at 30 min intervals. After that the cells were rinsed with 1X PBS and fixed with 4% paraformaldehyde/PBS (pH 7.4) for 10 min at room temperature. Then the cells were rinsed with 1X PBS and incubated with 0.5% Triton X-100/PBS containing 5 μg/mL 4′,6-diamidino- 2-phenylindole (DAPI) for 10 min. The coverslips were rinse with 1X PBS and mounted in glycerol-based mounting media on microscope slides. z stack microscopic images were acquired using a confocal laser scanning microscope (Zeiss) at 0.1–0.5 μm intervals with a 633 oil-immersion objective lens. Three-dimensional (3D) images of the mitochondria and nuclei were generated and analyzed using Imaris 9.6 software (Bitplane AG). 3D reconstructions were created using the Surfaces function with a smoothing surface detail of 0.198 μm, and thresholding by background subtraction (diameter of largest sphere of 0.2 μm) for mitochondria and by absolute intensity for nuclei. The morphologies of mitochondria were distinguished with sphericity index of the reconstructed surfaces: 0.85–1 (spherical/fragmented), 0.55–0.85 (fused/elongating) and 0–0.55 (tubular/network). The relative volume of mitochondria was calculated by dividing total mitochondria volume by total nuclei volume per image. At least 8 images per cell types were evaluated to calculate statistical significance.

#### Immunofluorescent staining

Immunofluorescent staining was performed as previously described.^83^ Briefly, gastruloids and PGCLC spheroids were fixed in 4% paraformaldehyde/PBS at 4°C overnight. Then the specimens were rinsed in 1X PBS and equilibrate in 30% sucrose/PBS at 4°C overnight. After that, the specimens were embedded in OCT compound and sectioned to 6–7 μm thickness using a cryostat. The sections were heated in 1X Target Retrieval Solution (pH 6.1) (Dako) and then incubated in blocking buffer containing 3% skimmed milk/PBST (0.1% Tween 20/PBS) for 1 h at room temperature. Then the sections were incubated with the following primary antibodies (1:100, R&D Systems) in PBST at 4°C overnight: goat anti-BRACHYURY, goat anti-SOX17 and goat anti-SOX2. The sections were rinsed in PBST and then incubated with secondary antibody conjugated with either Alexa Fluor 488 or −647 (1:500, Invitrogen) in PBST containing 5 μg/mL DAPI for 1 h at room temperature. After rinsing in PBST, the sections were mounted in glycerol-based mounting media and imaged using a confocal laser scanning microscope (Zeiss). For immunofluorescent staining of cultured cells, the cells were fixed in 4% paraformaldehyde/PBS for 15 min at room temperature. After rinsing in 1X PBS, the cells were blocked with 3% skimmed milk. Then the cells were permeabilized with 0.3% Triton X-/PBS (TPBS) and incubated with the following primary antibodies at 4°C overnight: goat anti-BRACHYURY (1:1000, R&D Systems), goat anti-SOX17 (1:1000, R&D Systems) and rabbit anti-TUBB3 (1:5000, Biolegend). After rinsing in TPBS, the cells were incubated with secondary antibody conjugated with either Alexa Fluor 488 or −647 (1:1000, Invitrogen) at 4°C overnight. On the next day, the cells were rinsed in TPBS and counterstained with DAPI. Then the cells were mounted in glycerol-based mounting media and imaged using a confocal laser scanning microscope (Zeiss). Images were processed using Adobe Photoshop.

#### Karyotype analysis

EpiLSC were incubated with 0.2 mg/mL KaryoMAX colcemid (Gibco) for 4 h in a 37°C 5% CO2 humidified incubator. Then the cells were trypsinized and collected by centrifugation. The cell pellet was resuspended with 4 mL 75 mM KCl solution and incubated at room temperature for 10 min. After centrifugation, the supernatant was removed and the pellet was resuspended with 4 mL of freshly prepared fixative solution (absolute methanol:glacial acetic acid (3:1)). This step was repeated twice, and the pellet was resuspended with 500 mL fixative solution. Then the cell suspension was dropped onto glass slides and air-dried. The metaphase chromosome spreads were stained with 4% KaryoMAX Giemsa (Gibco) in Gurr phosphate buffer (pH 6.8) for 5 min. After that, the slides were rinsed in distilled water and dehydrated through an ascending series of ethanols ending in xylene. The slides were mounted in DPX mountant (Sigma-Aldrich) and imaged using a bright-field microscope (Zeiss). The number of chromosomes from 20 randomly selected spreads were counted.

#### Fluorescence-activated cell sorting (FACS)

For culture cells, single cell suspension was prepared by dissociating cell colonies in 1X TrypLE express enzyme (Gibco) and followed by resuspending in FACS buffer (containing 0.1% BSA & 0.1 mg/mL DNase I). For PGCLC spheroids, single cell suspension was prepared as previously described.^84^ Briefly, the BVSC PGCLC spheroids were incubated in dissociation buffer (containing 1X TrypLE express enzyme & 0.1 mg/mL DNase I) at 37°C for 5–15 min. After centrifugation, the cell pellets were rinsed twice in FACS buffer and followed by holding in FACS buffer. Prior to cell sorting, the cells were stained with 1 μg/mL propidium iodide (PI) to exclude dead cells. Live single cells were sorted into 384-well plates using SH800 cell sorter (SONY) with 100 μm nozzle chip. Stella-CFP was excited with violet laser (405 nm; bandpass filter: 450/50), Blimp1-VENUS with blue laser (488 nm; bandpass filter: 525/50) and PI with yellow/green laser (561 nm; bandpass filter: 600/60). For Oct4-DE-EGFP PGCLC spheroids, the single cell suspension was stained with PE-conjugated anti-CD61 and eFluor660-conjugated anti-SSEA1 antibodies prior to FACS. Dead cells were excluded by staining with 0.1 μg/mL 4′,6-diamidino-2-phenylindole (DAPI). PE-CD61 was excited with yellow/green laser, eFluor660-SSEA1 with red laser (638nm; bandpass filter: 665/30) and DAPI with violet laser.

#### Energy metabolism assays

Energy metabolism assays were performed using Seahorse XFe96 analyzer (Agilent) as previously described following manufacturer’s instructions.^85^ Briefly, ESC, EpiLSC and EpiSC were seeded in gelatin-coated XF96 tissue culture microplates at densities of 2.5 × 10, 4.3×10^4^ and 3.5×10^4^ cells/well respectively, and cultured in a 37°C 5% CO2 humidified incubator for 24 h. On the next day, the media were replaced with XF base media (pH 7.4). For the Mitochondrial Stress Test assay, the XF base media were supplemented with 10 mM D-glucose, 1 mM sodium pyruvate and 2 mM L-glutamine, and oxygen consumption rate (OCR) and extracellular acidification rate (ECAR) were measured every seventh minute under basal conditions and after sequential addition of oligomycin (1 μM), carbonyl cyanide-4-(trifluoromethoxy) phenylhydrazone (FCCP) (1 μM) and antimycin A (2 μM)/rotenone (1 μM). Experiments were repeated at least twice and measurements were collected from 13 wells for each cell types per assay per experiment. After the assays, the cells were lysed in lysis buffer (containing 50 mM Tris-HCl (pH 7.4), 180 mM NaCl, 1 mM EDTA, 1% Triton X-100 & 15% glycerol), and protein concentration in the whole cell lysates were quantified using Pierce BCA protein assay kit (Thermo Scientific) and measured using a microplate reader (Molecular Devices). Both ECAR and OCR were normalized with protein content.

#### Bulk ATAC-seq library preparation

Bulk ATACseq was performed following an established protocol.^28^ Briefly, cells were fixed in formaldehyde (1% final concentration) for 10 min at room temperature and quenched with glycine (0.125 M final concentration). Then the cells were rinsed twice with 1X PBS. 50,000 cells were lysed in 50 μL lysis buffer (containing 10 mM Tris-HCl (pH 7.4), 10 mM NaCl, 3 mM MgCl2, 0.1% Igepal CA-630). The harvested nuclei were tagmented in 50 μL transposase reaction mix (containing 1x TD buffer and 100 nM Tn5 transposase) at 37°C for 30 min. Then 50 mL of reverse crosslink solution (containing 100 mM Tris-HCl, 2 mM EDTA, 2% SDS, 0.4 M NaCl) and 1 μL 20 mg/mL proteinase K were added to the mixture and incubated overnight at 65°C with 1200 rpm shaking. The tagmented DNA fragments were purified using Qiagen MinElute kit. The library was amplified in 50 μL PCR reaction mix (containing 1X NEBnext high fidelity PCR master mix and 1.25 μM of Nextera PCR primer 1 and 2) at the following PCR conditions: 72°C for 5 min; 98°C for 30 s; and thermocycling at 98°C for 10 s, 63°C for 30 s, 72°C for 1 min. The cycle number was determined by qPCR in order to terminate the amplification before saturation. In this study, we used a total of 9–13 PCR amplification cycles. The library was purified with SPRI beads at 1:1 ratio. Two experimental replicates per cell type were sequenced using Illumina NovaSeq 6000 system.

#### Single-cell RNAseq library preparation

Sequencing libraries for scRNA-seq were generated following the Smart-seq3 protocol.^86^ Briefly, live single cells were FACS-sorted into 3 μL lysis buffer/well (containing 0.5 μM oligoT30VN, 0.5 mM/each dNTPs, 5% PEG, 0.1% Triton X-100 & 0.4 U/μL RRI) of 384-plates. The plates were incubated at 72°C for 10 min before 1 μL of reverse transcription mix (containing 30 mM Tris-HCl (pH8.3), 40 mM NaCl, 1mM GTP, 2.5 mM MgCl2, 8 mM DTT, 1 U/μL RRI, 2 μM N8_TSOs & 2 U/μL Maxima H-minus reverse transcriptase) was added to each well. Reverse transcription and template switching were performed at 42°C for 90 min, followed by 10 cycles at 50°C for 2 min and 42°C for 2 min, and terminated at 85°C for 5 min cDNA amplification was performed by adding 6 μL of PCR mix was added to each well (with final concentration of 1X KAPA HiFi PCR buffer, 0.1 mM/each dNTPs, 0.1 μM forward primer, 0.1 μM reverse primer & 0.02 U/μL HiFi DNA polymerase), at the following PCR conditions: 98°C for 3 min, 20 cycles at 98°C for 20 s, 65°C for 30 s, 72°C for 4 min, and followed final extension at 72°C for 5 min. The amplified cDNAs were purified using 22% PEG beads at 0.6:1 (bead:sample) ratio. cDNA libraries were quality checked using Bioanalyzer and quantified using QuantiFluor dsDNA System. Tagmentation was performed in 2 μL reaction mix (containing 200 pg cDNAs, 1X tagmentation buffer, 0.2 μL ATM Tn5) at 55°C for 10 min. Then, 0.5 μL 0.2% SDS was added to each well and incubate at room temperature for 5 min. Then 1.5 μL Nextera XT index primers (0.5 μM/each) and 3 μL PCR mix (containing 1X Phusion buffer, 0.2 mM/each dNTPs & 0.01 U/μL Phusion DNA polymerase) were added to each well and incubated at the following PCR conditions: 72°C for 3 min, 98°C for 3 min, 12 cycles at 98°C for 10 s, 55°C for 30 s, 72°C 30 s, and followed by final extension at 72°C at 5 min. The tagmented libraries were pooled and purified using 22% PEG beads at 0.7:1 (bead:sample) ratio. Purified libraries were quality checked using Bioanalyzer and sequenced using NovaSeq 6000 platform at 150-bp paired end.

### QUANTIFICATION AND STATISTICAL ANALYSIS

#### RT-qPCR

Total RNAs were isolated with RNeasy Mini Kit (Qiagen) and cDNAs were synthesized using oligo(dT) primer and SuperScript II reverse transcriptase (Invitrogen) following the manufacturer’s instructions. RT-qPCR was performed with biological and technical duplicates using PowerUp SYBR green master mix (Applied Biosystems) and StepOnePlus real-time thermal cycler (Applied Biosystems). Target gene expression was normalized with the expression of a housekeeping gene *Ppia* (*peptidylproly isomerase A*). Primer sequences are listed in Table S6.

#### Alignment and peak calling of ATAC-seq sequencing reads

Paired-end sequencing reads were cleaned with adapter removal by pyadapter_trim.py from the PEPATAC pipeline.^69^ Reads were mapped to mm10 reference genome via bowtie2 with parameter –very-sensitive. Mitochondrial reads and PCR duplicates were removed. After alignment, we used the PEPATAC pipeline to check for TSS enrichment and fragment length contribution to confirm the characteristics of ATAC-seq libraries. Filtered paired reads were corrected for the Tn5 cutting sites shifts with +4 bases for positive strand and –5 bases for negative strand. All mapped reads were extended to 50 bp centered by Tn5 offset. Next, peak calling was performed by MACS2 with –nomodel –shift 0. Irreproducible discovery rate (IDR) analysis was performed and peaks that fails threshold of 5% were removed.^87^ Bigwig files were generated with the value of fold change compared to the background. The visualization was achieved by IGV tool.^71^ The intensities of peaks were calculated by Deeptools.^72^ Heatmap and aggregate signal of peak regions were plotted by Deeptools. Motif enrichment for selected peak set was performed with findmotifs.pl from Homer.^88^

#### Differential peak clustering

ATAC-seq peaks from all the samples were merged and reduced for overlapping regions to form the peak set. Raw reads within the peak set were normalized by depth using edgeR.^73^ Pearson correlation and PCA were performed on the Log normalized counts of the peak set which includes all the IDR peaks of the samples. TCseq (https://doi.org/10.18129/B9.bioc.TCseq) was used to obtain differential peaks between any of the two from ESCs, EpiLSCs, d2 EpiLCs and EpiSCs with log2(fold change) > 1 and false discovery rate (fdr) < 0.05. Then the differential peaks were clustered with fuzzy k means algorithm by TCseq. Peaks with likelihood <0.8 for cluster identification were removed.

#### Peak annotation and GO analysis

ATAC peaks were annotated by assigning them to the nearest genes within the same topological associated domain (TAD). It was shown that TAD remain stable among different cell types. Therefore, TAD boundaries identified in ESCs were used.^89^ Pathway enrichment analysis for the annotated genes was performed with Metascape.^74^

#### Single-cell alignment and processing

Sequencing reads were mapped to mm10 and counted with zUMIs^75^ pipeline with default settings for smart-seq^86^ as previously reported. For smart-seq3, UMI counts were used. For smart-seq2, all counts were used. Seurat V3^34^ was used for downstream processing unless otherwise stated. First, we performed quality control to remove cells with low number of genes and counts according to Seurat violin plots and removed cells with greater than 10% mitochondrial content. Then, using default parameters, the expression matrix was Log normalized and scaled. Dimension reduction by PCA was performed with 3000 variable genes. Batch effects were removed by Harmony.^38^ The number of PCs and harmony dimensions used for cell clustering and UMAP embedding were decided by Elbowplot which identifies an elbow in the graph. In silico bulk RNAseq used in Figure 6C was generated by aggregating the single cells’ expression profile using Seurat V3. Pseudtotime trajectory analysis was performed by Slingshot.^76^ For Slingshot dimension reduction, the data were not scaled by the variance because genes are considered as not equally informative in trajectory. Differentially expressed genes between groups or clusters were identified by MAST^78^ with log2(fold change) > 1 and adjusted p value < 0.05. Consecutively differentially expressed genes in FTW, EpiLSC and FS (Table S4) were identified with log2(fold change) > 0.58 and adjusted p value < 0.05 both in FTW vs EpiLSC and EpiLSC vs FS comparison. Pseudotime associated differentially expressed genes were identified by TradeSeq^77^ with fdr<0.05. Cell cycle was analyzed by CellCycleScoring in Seurat. Cell entropy was analyzed with SCENT.^48^

#### Single-cell stage label transfer (scSTALT)

Integrating PSCs from different laboratories using current methodologies led to over-integration due to the lack of common cells in different cell lines. Since the PSCs we studied presumably fall in the trajectory from naive to primed differentiation, we reasoned that with information of dynamic gene expression along the trajectory as a reference, we can produce continuously binned pseudotime points as in-silico anchors for assigning query cells to the pseudotime as well as integrating datasets even without representative cell types. Therefore, we developed Single-cell stage label transfer (scSTALT) which briefly involves three steps.

##### pseudotime inference

First, a pseudotime trajectory of the reference dataset should be generated. Here we used the method Slingshot and one can adapt alternative TI method as their own study required. As suggested by Slingshot, the dimension reduction we used is PCA on non-scaled log normalized gene expressions since the importance of each gene in influencing the trajectory differs. After the pseudotime was obtained, we normalize the pseudotime within the range of 0–1.

##### kernel gene expression profile

It has been proposed that cell stages can be identified by the expression of the dynamic genes which are preserved along all states of the cell. Successfully identifying these core genes allows one to differentiate between cell states. In practice, TradeSeq^77^ infers trajectory associated differentially expressed genes with smooth functions for the gene expression measures along pseudotime for each lineage using generalized additive models. Therefore, with TradeSeq, we can obtain a kernel gene expression profile (k genes) which smoothed the biological process into a fixed model which includes m stages. Therefore, we use this kernel gene profile as the trajectory reference comprising m stages and has k essential dynamic genes as expressed in the following expression:

$$\text{R}\left(\text{k,m}\right)=\left[\begin{array}{ccc} g_{1}^{1}\;\;g_{2}^{1} & \ldots & g_{m-1}^{1}\;\;g_{m}^{1}\\ \vdots \vdots & \ddots & \vdots \vdots \\ g_{1}^{k}\;\;g_{2}^{k} & \cdots & g_{m-1}^{k}\;\;g_{m}^{k} \end{array}\right]$$

##### Pseudotime label transfer and data integration

For the query dataset with n cells, we retrieve their k gene expression profile and therefore obtain the following data matrix:

$$\text{E}\left(\text{k,n}\right)=\left[\begin{array}{ccc} g_{1}^{1}\;\;g_{2}^{1} & \ldots & g_{n-1}^{1}\;\;g_{n}^{1}\\ \vdots \vdots & \ddots & \vdots \vdots \\ g_{1}^{k}\;\;g_{2}^{k} & \cdots & g_{n-1}^{k}\;\;g_{n}^{k} \end{array}\right]$$

Then the issue is how to project each single cell in matrix E to the stages in R (in silico anchors). We found that in this circumstance, the metric Euclidean distance is not robust to batch effect. In contrast, cosine similarity is a measure of similarity between two sequences of numbers and works well in simulated as well as real datasets. Therefore, we used cosine similarity to assign each cell to which it has. Specifically, for each cell (E[j]) in the query,

$$E\left[j\right]=\left[g_{1}^{j},g_{2}^{j},\ldots ,g_{k-1}^{j},g_{k}^{j}\right]\;\;j\in \left\{1,2,\ldots ,n\right\}$$

we calculated their cosine similarity to each cell (R[i]) in the kernel reference

$$R\left[i\right]=\left[g_{1}^{i},g_{2}^{i},\ldots ,g_{k-1}^{i},g_{k}^{i}\right]\;\;i\in \left\{1,2,\ldots ,m\right\}$$

using the equation as below:

$$CS\left(A,B\right)\;\;\;:\;\;\;\;=\;\;\;\cos\left(\theta \right)\;\;=\;\frac{A\cdot B}{\left\|A\right\|\;\;\;\left\|B\right\|}\;\;=\;\;\frac{\sum_{i=1}^{n}A_{i}B_{i}}{\sqrt{\sum_{i=1}^{n}A_{i}^{2}}\sqrt{\sum_{i=1}^{n}B_{i}^{2}}}$$

Then we obtained the highest cosine similarity and assign the query cell to the corresponding stage.


$$S\left(j\right)=\sum_{i=1}^{m}\max\left(CS\left(E\left[j\right],R\left[i\right]\right)\right)\;\;\;\;j\in \left\{1,2,\ldots ,n\right\},i\in \left\{1,2,\ldots ,m\right\}$$

Subsequently, the cell j in the query will be labeled the pseudotime or cellular stage to whom is has the largest cosine similarity S(j). One can also determine if the query cell belongs to the reference cell type depending on cutoff of the S(j) value.

One superiority of this method is that one can accurately learn the cellular states of a given dataset regardless of whether it is suitable to adopt de novo TI method such as Slingshot.^76^ Another advantage of our method is the computation speed. With the kernel gene expressions ready, the pseudotime transferring is ultrafast. Through the implement in R, we used a randomly generated kernel of 500 genes and 100 timepoints, it just took several seconds to minutes to assign identity for thousands to several tens of thousands of cells with a 2.8 GHz Processor, 16 G RAM.

Additionally, since the pseudotime transferring harmonized gene expressions regardless of batches, our use of scSTALT could fulfill data integration. Instead of scaling through mutual nearest neighbors or shared populations between different batches, we assign cells into time-series partitions (in Figure 3E we used 100 partitions) and then scale the average gene expressions for each partition. After the batch correction, the cells of two batches are aligned in the order of their cellular stage regardless of the batches compared. Even though the reference and query cells are mosaic regarding their differentiation stages, they can be anchored into the same pseudotime time point in the kernel gene profile and therefore avoid overfitting as illustrated in the benchmarking in Figures 3D and 3E.

#### Data simulation

To test our method scSTALT, we generated simulated dataset which we have the prior knowledge of their cellular differentiation stages. We used R package Splatter with parameter Step = True to generate continuously differentiating trajectories. Briefly, we simulated a scenario with two batches of 3000 cells containing varying stages within the differentiation process. The differentiation process was designed to consist of cells from stage 1 to stage 100. We then generate one batch consists of cells from varying stages, including stage 1:20 (designated as parturition A), stage 40:60 (designated as parturition C) and stage 80:100 (designated as parturition E), whereas the other batch consists of cells from stage 30:35 (designated as parturition B) and 70:75 (designated as parturition E). With ten repetitions of different combination of signal to noise ratio (0.05, 0.1, 0.15, 0.2, 0.25) and non-linear gene proportions (0.1, 0.2, 0.3, 0.4, 0.5), we have in total generated 250 simulations. However, when the signal to noise ratio is too low (0.05) and the non-linear gene proportion is too high (0.5), all methods failed. These simulations were therefore excluded for the evaluation.

#### Evaluation

To evaluate the accuracy of the stage-transferring, we chose R^2^ coefficient to measure the goodness of the fitting. R^2^ score is a statistical measure evaluating the predictions, with value 1 indicating predictions approximating the real values. It can evaluate differences between stage predicted (pseudotime) and the stage observed (stage in the simulation). We used R package MLmetrics (http://github.com/yanyachen/MLmetrics) to compute R^2^ coefficient.

#### Single-cell WGCNA

The package of high dimension WGCNA (hdWGCNA)^41^ is used for single-cell WGCNA analysis. First, metacells are constructed by the k-Nearest Neighbors (KNN) algorithm. Gene modules were then identified by ConstructNetwork with soft_powere = 10. Genes were designated to the module by using the initially assigned gene module. Hub genes were identified as the most connected genes within each module.

#### Activities of gene sets in single cell

For the WGCNA gene module, the entire module activities were estimated by the WGCNA metric Module Eigengenes (MEs).^90^ MEs of single cells are computed by performing principal component analysis (PCA) on the corresponding gene expression matrix for the module genes. The first PC of each of these PCA matrices is the MEs. For the naive, formative, primed gene set, the metabolic pathway gene sets and the XaXa v.s. XaXi PGCLC highly expressed gene signature, AUCell was used to identify the transcriptional activities of each cell.^58^ Naive, formative and primed gene sets were defined by uniquely up-regulated genes in ESC 2i, d2 EpiLCs and EpiSCs respectively. AUCell uses the ‘‘Area Under the Curve’’ (AUC) to calculate the relative expression of the gene set. The scoring method is ranking-based. A higher score indicates larger relative transcriptional activities. The metabolic pathway activity estimation was performed using scMetabolism^42^ with the method being AUCell.

#### RNA velocity

Velocyto^79^ was used to estimate the unspliced and spliced transcripts for each gene from the alignment bam files. ScVelo^47^ was then used to model gene velocities and study cellular dynamics. The cells were visualized using dimension reductions of harmony embedding.

#### Statistical analyses

The transcription factor motif enrichment was performed with cumulative hypergeometric test. Functional pathway enrichment was performed with hypergeometric test. Differentially expressed genes were identified via generalized regression model fitting and tests with asymptotic χ^2^ null distributions. Additionally, paired sample t test was used to determine the significance for ATAC-Seq peak differences between samples. Two-tailed Student’s t test was used to determine the significance of difference between the control the experimental groups for the rest. Statistical significance was defined by p < 0.01 (**) and p < 0.001 (***). The p value and test of each comparison was indicated in the result or figure legends. The sample numbers were indicated in the figure legends. The mean and SD were indicated in figures. No method was used to determine whether the data met assumptions of the statistical approach.

## Supplementary Material

1

2

3

4

5

6

7

## Figures and Tables

**Table:** KEY RESOURCES TABLE

REAGENT or RESOURCE	SOURCE	IDENTIFIER
Antibodies		

Human/Mouse Brachyury antibody	R&D Systems	Cat# AF2085; RRID:AB_2200235
Human/Mouse Sox17 antibody	R&D Systems	Cat# AF1924; RRID:AB_355060
Human/Mouse/Rat Sox2 antibody	R&D Systems	Cat# AF2018; RRID:AB_355110
Human/Mouse Oct3/4 antibody	R&D Systems	Cat# AF1759; RRID:AB_354975
Human/Mouse Tubb3 antibody	BioLegend	Cat# 802001; RRID:AB_2564645
PE anti-mouse/rat CD61 antibody	BioLegend	Cat# 104307; RRID:AB_313084
eFlour 660 anti-human/mouse SSEA1 antibody	Thermo Fisher Scientific	Cat# 50-8813-42; RRID:AB_11219681
Donkey anti-goat IgG (H + L) cross-adsorbed secondary antibody, Alexa Fluor 488	Thermo Fisher Scientific	Cat# A-11055; RRID:AB_2534102
Donkey anti-goat IgG (H + L) cross-adsorbed secondary antibody, Alexa Flour 647	Thermo Fisher Scientific	Cat# A-21447; RRID:AB_2535864

Chemicals, peptides, and recombinant proteins		

MitoTracker Red CMXRos	Invitrogen	Cat# M-7512
KaryoMax Colcemid	Gibco	Cat# 15212012
KaryoMax Giemsa stain	Gibco	Cat# 10092013
CHIR99021	PeproTech	Cat# 2520691
PD0325901	PeproTech	Cat# 3911091
PD173074	PeproTech	Cat# 2191178
Recombinant Mouse LIF	Merck Millipore	Cat# ESG1107
Recombinant Human bFGF	Gibco	Cat# 13256029
Recombinant Human Activin A	PeproTech	Cat# 120-14P
Human Plasma Fibronectin	Merck Millipore	Cat# FC010
Recombinant Human BMP4	R&D Systems	Cat# 314-BP-050
Recombinant Human BMP8a	R&D Systems	Cat# 1073-BP-010
Recombinant Mouse SCF	R&D Systems	Cat# 455-MC-010
Recombinant Mouse EGF	R&D Systems	Cat# 2028-EG-200

Critical commercial assays		

Alkaline Phosphatase Detection Kit	Sigma-Aldrich	Cat# SCR004
Nextera XT DNA Library Preparation Kit	Illumina	Cat# FC-131-1096

Deposited data		

ATAC-Seq of ESCs and EpiSCs	Bleckwehl et al., 2021^29^	GSE155058
ATAC-Seq of FS	Kinoshita et al., 2021^19^	GSE131556
ATAC-seq of FTW, EpiLCSC, EpiLC and EpiLC + CHIR	This paper	PRJNA856446
scRNA-Seq of FTW, EpiLCSC, EpiLC and EpiLC + CHIR	This paper	PRJNA856446
scRNA-Seq of ESCs, d1-d3 EpiLCs and EpiSCs	Bleckwehl et al., 2021^29^	GSE155088
scRNA-Seq of E4.5-E6.5 epibalst	Mohammed et al., 2017^39^	GSE100597
scRNA-Seq of E4.5-E6.5 epibalst	Argelaguet, et al., 2019^31^	GSE121708
scRNA-Seq of FS	Kinoshita et al., 2021^19^	GSE156589
scRNA-Seq of fPSC	Wang et al., 2021^20^	GSE154290
RNA-Seq of E4.75 and E5.0 epiblast	Shahbazi, et al., 2017^40^	E-MTAB-5147
RNA-Seq of PGCLC	Ishikura, et al., 2021^53^	GSE168222
scRNA-Seq of PGC	Grosswendt, et al., 2020^54^	GSE137337
RNA-Seq of PGC	Hill, et al., 2018^56^	GSE76973
RNA-Seq of PGC	Yamaguchi, et al., 2013^55^	GSE41908
scRNA-Seq of PGCLC	This paper	PRJNA856446
RNA-Seq of female PGCLC	Severino, et al., 2022^57^	GSE169201

Experimental models: Cell lines		

Mouse: BVSC mESCs	Ohinata et al., 2008^23^; Ohta et al., 2017^23^	N/A
Mouse: BVSC sEpiLCs	This study	N/A
Mouse: BVSC EpiSCs	This study	N/A
Mouse: BVSC FTW-ESCs	Yu et al., 2021^18^	N/A
Mouse: Oct4-DE-EGFP mESCs	Wu et al., 2015^52^	N/A
Mouse: Oct4-DE-EGFP sEpiLCs	This study	N/A

Oligonucleotides		

All primers used in this study are listed in Table S6	N/A	N/A

Software and algorithms		

GraphPad Prism 8	GraphPad Prism	https://www.graphpad.com
Imaris 9.9.0	Bitplane AG	https://imaris.oxinst.com
scSATLT	This paper	https://github.com/DengLab-KI/scStalt
PEPATAC pipeline	Smith et al., 2021^69^	http://pepatac.databio.org/en/latest/
MACS2	Zhang et al., 2008^70^	https://hbctraining.github.io/Intro-to-ChIPseq/lessons/05_peak_calling_macs.html
IGV	Robinson et al., 2011^71^	https://software.broadinstitute.org/software/igv/home
Deeptools	Ramírez et al., 2016^72^	https://deeptools.readthedocs.io/en/develop/content/list_of_tools.html
edgeR	Robinson, et al., 2010^73^	https://bioconductor.org/packages/release/bioc/html/edgeR.html
TCseq	https://doi.org/10.18129/B9.bioc.TCseq	https://bioconductor.org/packages/release/bioc/html/TCseq.html
Metascape	Zhou, et al., 2019^74^	https://metascape.org/gp/index.html#/main/step1
zUMIs	Parekh, et al., 2018^75^	https://github.com/sdparekh/zUMIs
Seurat v3	Hao, et al., 2021^37^	https://satijalab.org/seurat/index.html
Harmony	Korsunsky, et al., 2019^38^	https://github.com/immunogenomics/harmony
FastMNN	Haghverdi, et al., 2019^35^	https://bioconductor.org/packages/release/bioc/html/batchelor.html
Splatter	Zappia, et al., 2019^36^	https://bioconductor.org/packages/release/bioc/html/splatter.html
Slingshot	Street, et al., 2018^76^	https://github.com/kstreet13/slingshot
TradeSeq	Berge et al., 2020^77^	https://github.com/statOmics/tradeSeq
SCENT	Che, et al., 2019^48^	https://github.com/aet21/SCENT
MAST	Finak, et al., 2015^78^	https://www.bioconductor.org/packages/release/bioc/html/MAST.html
hdWGCNA	Morabito, et al., 2021^41^	https://github.com/smorabit/hdWGCNA
AUCell	Aibar, et al., 2017^58^	https://bioconductor.org/packages/release/bioc/html/AUCell.html#:~:text=AUCell%20allows%20to%20identify%20cells,expressed%20genes%20for%20each%20cell.
scMetabolism	Wu, et al., 2021^42^	https://github.com/wu-yc/scMetabolism
Velocyto	Manno, et al., 2018^79^	http://velocyto.org/
scVelo	Bergen, et al., 2020^47^	https://scvelo.readthedocs.io/VelocityBasics/

## Highlights

- EpiLSCs are molecularly distinct from other formative pluripotency stem cells
- Single-cell stage label transfer (scSTALT) enables integrated pseudotime analysis
- WNT activation sustains metastable formative pluripotency of EpiLSCs
- EpiLSCs have competence for germline induction with enhanced maturation by PD
