# Supplementary material for: Epiblast-like stem cells established by Wnt/β-catenin signaling manifest distinct features of formative pluripotency and germline competence
Source: Cell Rep. Author manuscript; Available in PMC 2023 Mar 20. (PMC10026833; doi:10.1016/j.celrep.2023.112021)
Supplement: 1 [file NIHMS1870546-supplement-1.pdf]

Cell Reports, Volume *42*

Supplemental information

Epiblast-like stem cells established by Wnt/β-catenin signaling manifest distinct features of formative pluripotency and germline competence

Qing Luo, Han-pin Pui, Jiayu Chen, Leqian Yu, Paulo R. Jannig, Yu Pei, Linxuan Zhao, Xingqi Chen, Sophie Petropoulos, Jorge L. Ruas, Jun Wu, and Qiaolin Deng

Figure S1, Related to Figure 1

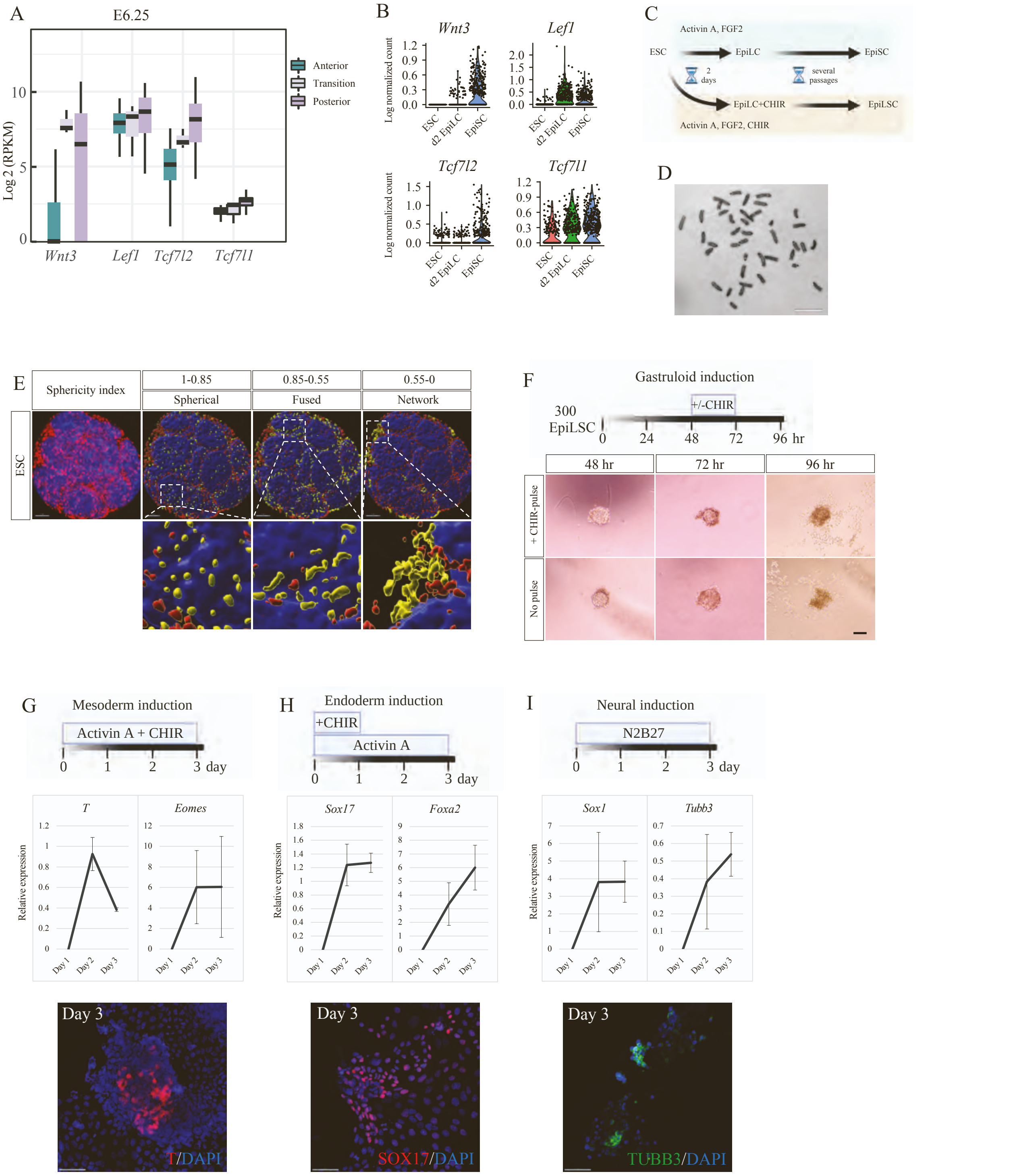

Figure S1. Characterization of EpiLSC via karyotype, mitochondrial and differentiation examination, Related to Figure 1

A, scRNA expression of *Wnt3*, *Lef1*, *Tcf7l2* and *Tcf7l1* in E6.25 epiblasts.

B, scRNA expression of *Wnt3*, *Lef1*, *Tcf7l2* and *Tcf7l1* in ESC, EpiLC and EpiSC.

C, Schematic diagram showing the generation of EpiLC, EpiLC+CHIR, EpiLSC and EpiSC from ESC.

D, Representative metaphase chromosome spread of EpiLSC at passage 20. Scale bar = 10 μm.

E, Determination and categorization of mitochondrial morphologies based on sphericity index using ESC as an example. Immunofluorescent staining of mitochondria in ESC with MitoTracker Red. Nuclei were counter-stained with DAPI. Right panels show Imaris 3D renderings of mitochondria (red) and nuclei (blue) in the cells. Mitochondria belong to each category are highlighted in yellow. Scale bar, 7 μm.

F, Gastruloid induction from EpiLSC using 300 cells with/without CHIR-pulse at 48 hr. Bright-field images of EpiLSC undergoing gastruloid assay using 300 cells with/without CHIR-pulse at 48 hr. Scale bar, 100 μm.

G-I, Induction of mesodermal, endodermal and neural lineages from EpiLSC. RT-qPCR analysis of mesodermal genes (*T* and *Eomes*), endodermal genes (*Sox17* and *Foxa2*) and neural genes (*Sox1* and *Tubb3*). Error bars represent SD from two independent experiments. Immuno-staining at Day 3 of induction. Nuclei were counter-stained with Dapi. Scale bar, 50 μm.

Figure S2, Related to Figure 2

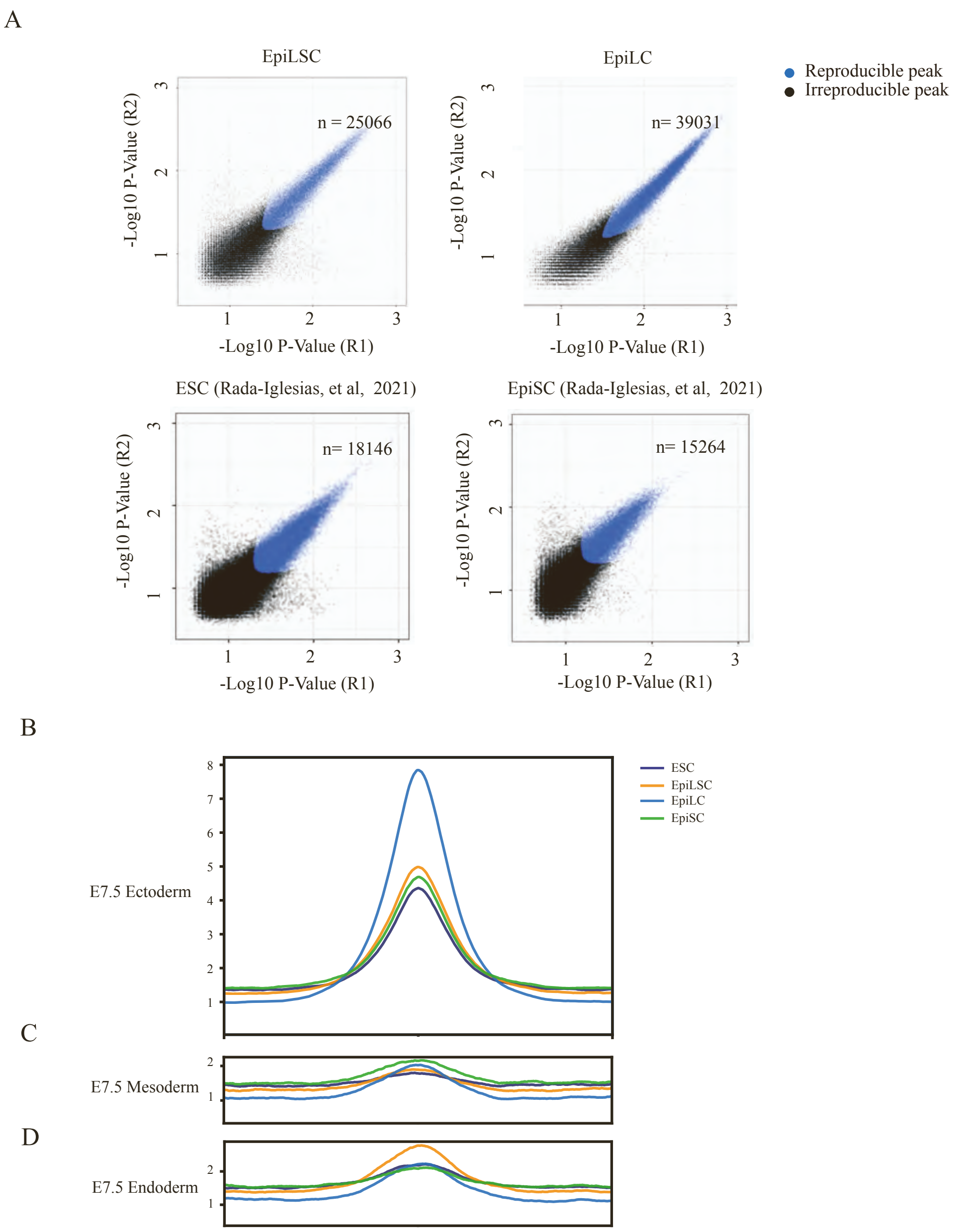

Figure S2. ATAC-Seq reproducible peak and lineage enhancer enrichment, Related to Figure 2

A, ATAC-Seq Reproducible peak number of EpiLC, EpiLSC, ESC and EpiSC.

B-E, Aggregate chromatin openness of the EpiLC, EpiLSC, ESC and EpiSC in E7.5 ectoderm (B), mesoderm (C), endoderm (D). The E7.5 three germ layer enhancers were identifed by anti-H3K27ac Chip-Seq.

Figure S3, Related to Figure 3

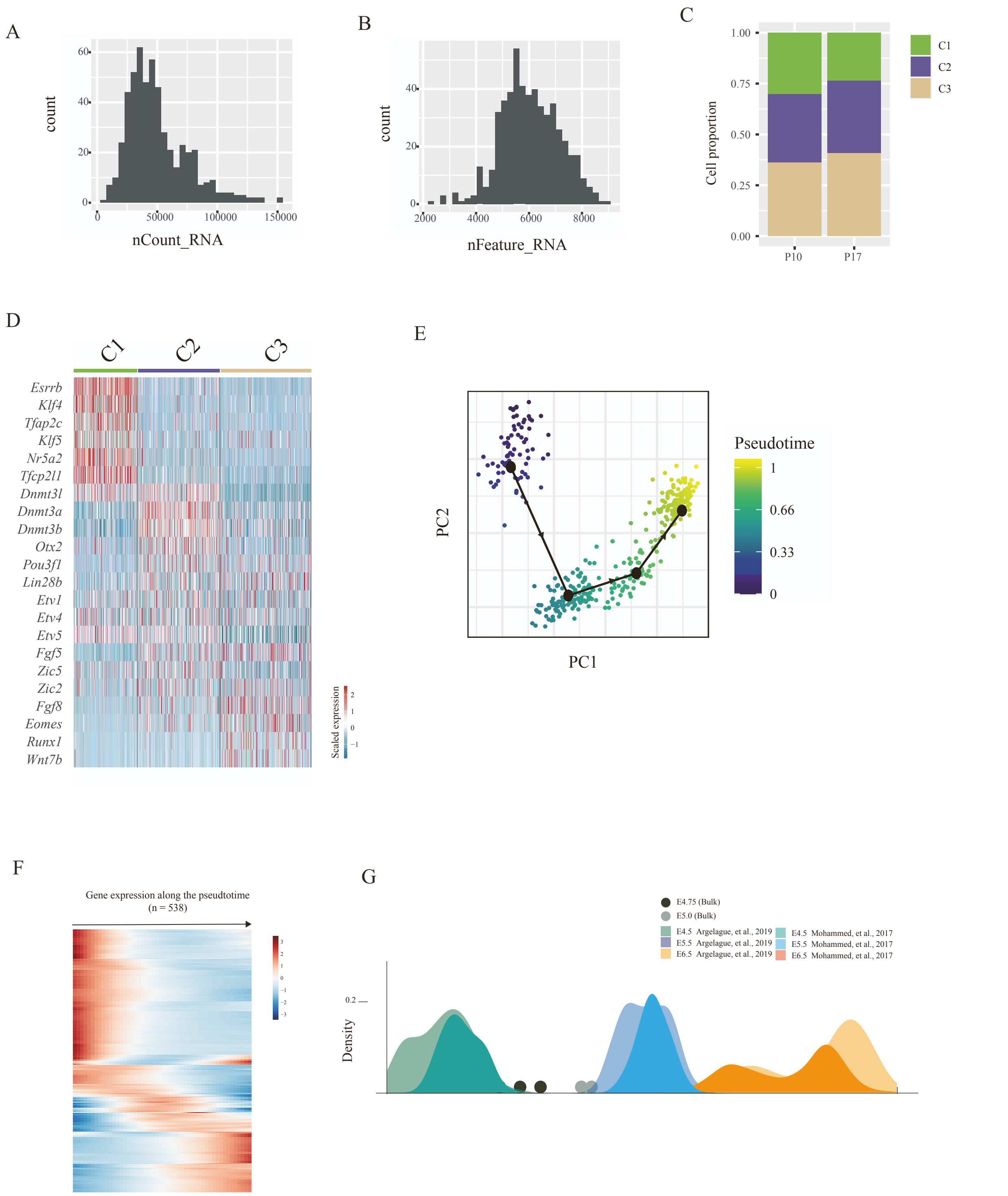

Figure S3. Single-cell RNAseq analysis of EpiLSC and buiding in vivo trajectory of E4.5-E6.5 epiblasts, Related to Figure 3

A, UMI and gene count (B) of EpiLSC single-cell sequencing.

C, Proportion of the three clusters in EpiLSC passage 10 and 17.

D, Heatmap showing the double naive, formative and primed markers in EpiLSCs.

E, Pseudotime trajectory of E4.5, E5.5 and E6.5 epiblast in the same dimension as in Figure 3G.

F, Heatmap of dynamic genes associated with the pseudotime trajectory in B. Some highlited genes are simultaneously shown in Figure 3G.

G, Pseudotime projection of bulk and single-cell RNA-Seq of E4.5-E6.5 epiblast cell into the referece built in Figure 3F. The reference was built with single-cell RNA-Seq of E4.5-E6.5 epiblast from Reik 2017 et.al. And the single-cell RNA-Seq of E4.5-E6.5 epiblast from another paper were projected approxiamtely to the corresponding time. Meanwhile, bulk RNA-Seq of E4.75 and E5.0 epiblasts were projected between E4.5 and E5.0.

Figure S4, Related to Figure 4

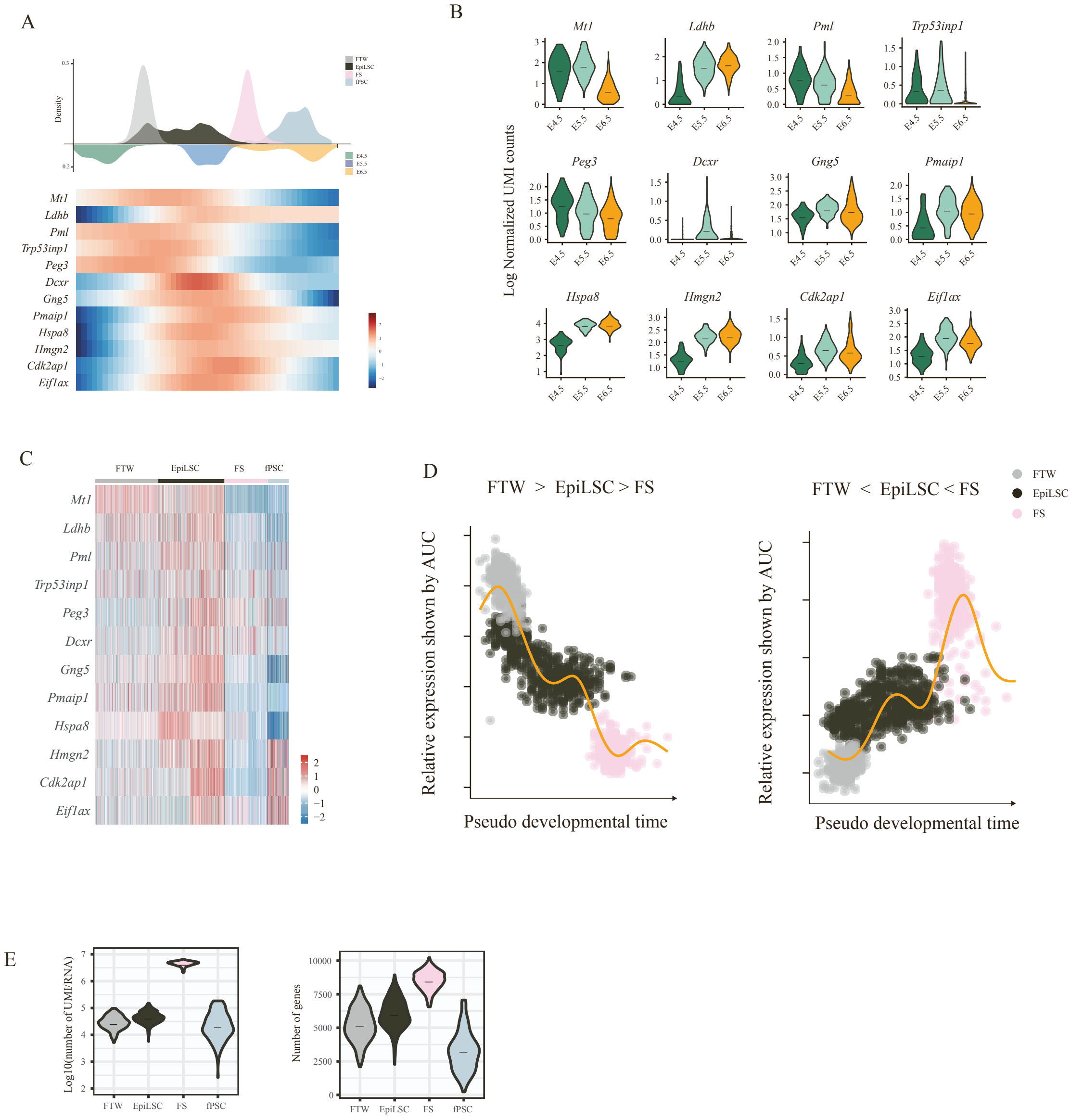

Figure S4. Single-cell RNAseq comparison among FTW, EpoiLSC, FS and fPSC, Related to Figure 4

A, Heatmap of E5.5 sepecific highly expressed genes are expressing in the time window overlapping with EpiLSC in the pseudotime scale.

B, Violin plot showing that the genes in A are highly expressed in E5.5 compaed to E4.5 and E6.5 epiblast.

C, Heatmap showing the expressions of genes in A in FTW, EpiLSC, FS and fPSC. These genes are highly expressed in EpiLSC compared to the other cells.

D, The expression activity of the gene sets defined by the genes of of FTW > EpiLSC > FS in FTW and vice versa. The expression activity was estimated by AUCell. Higher AUC value indicates higher gene set activity. The cells were plot in the x axis according to their projection into the pseudotime scale in Figure 3H. The result showed decreasing or increasing gene set activity in the sequenc of FTW, EpiLSC and FS for the consecutively down-regualting and up-regulating genes.

E, Log normalized UMI and gene count of FTW, EpiLSC, FS and fPSC single-cell sequencings.

Figure S5, Related to Figure 5

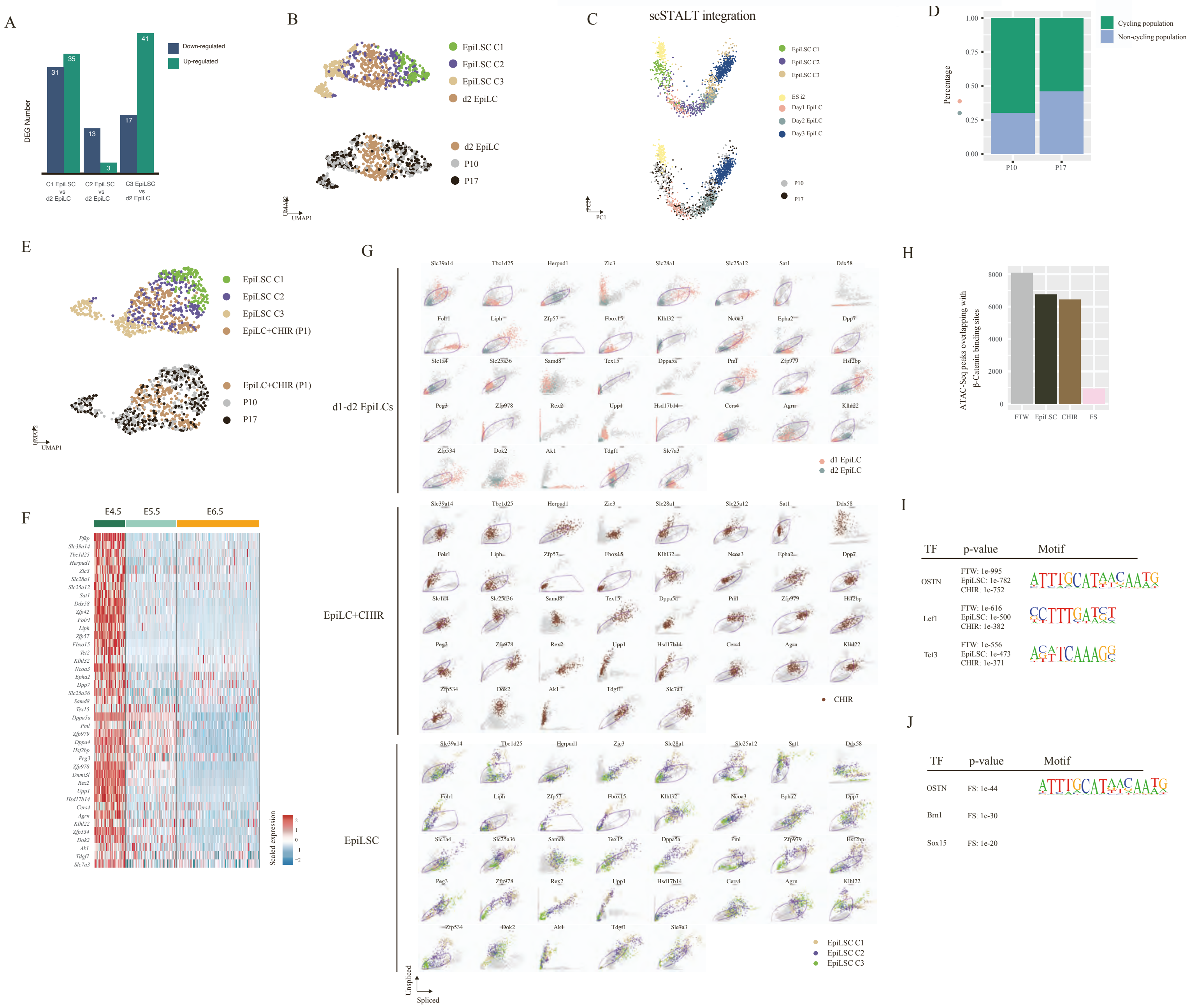

Figure S5. WNT/β-catenin signaling sustains dynamic cell states in EpiLSCs, Related to Figure 5

A, Numbers of differentially expressed genes in C1, C2 and C3 EpiLSC compared to d2 EpiLC.

B, Embedded integration of d2 EpiLC and EpiLSCs by scSTALT, showing the three clusters (upper) and passages (lower) of EpiLSCs.

C, Embedded integration of ES 2i, EpiLCs and EpiLSCs by scSTALT as in Figure 5A, showing the three clusters (upper) and passages (lower) of EpiLSCs. Passage 10 and 17 are similarly scattered in the embedding of the one used for velocity analysis as in Figure 5A.

D, Passage 10 and 17 had comparable proportion of cycling cell populations.

E, Embedded integration of EpiLC+CHIR and EpiLSCs by scSTALT, showing the three clusters (upper) and passages (lower) of EpiLSCs.

F, Heatmap of expression of the genes in Figure 5F in E4.5-E6.5 epiblast.

G, Velocities of the genes in Figure 5F which were not shown in Figure 5G. Here we highlighted the d1, d2 EpiLC, CHIR and C1-C3 EpiLSCs.

H, The number of ATAC-Seq peak overlapping with β-catenin binding sites in FTW, EpiLSC and CHIR and FS. The number of FTW is >8000, of EpiLSC and CHIR is >6000 and of FS is ~1000.

I, The top three enriched motifs for the ATAC-Seq peak overlapping with β-catenin binding sites as in E for FTW, EpiLSC and CHIR are simultinanously OSTN, Lef1 and Tcf3, whereas the top three for FS were OSTN, Brn1 and Sox15 (J).

Figure S6, Related to Figure 6

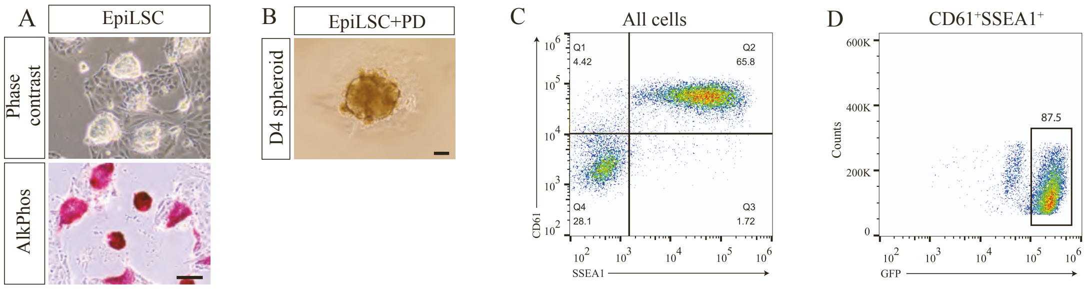

Figure S6. PGCLC inducetion from EpiLSC derived from Oct4-DE-EGFP mESC, Related to Figure 6

A, Representative culture morphology of EpiLSC derived from Oct4-DE-EGFP mESC. The upper and lower panels are phase contrast image and alkaline phosphatase staining of the culture. Scale bar, 100 µm.

B, The morphology of spheroids at Day 4 of PGCLC induction with PD supplementation. Scale bar, 100 µm.

C, FACS analysis of dissociated Day 4 spheroids stained with anti-CD61 and anti-SSEA1 antibodies.

D, The CD61 and SSEA1 double positive cells were also Oct4-DE-EGFP positive..
